# Weighted Betweenness Preferential Attachment: A New Mechanism Explaining Social Network Formation and Evolution

**DOI:** 10.1038/s41598-018-29224-w

**Published:** 2018-07-18

**Authors:** Alexandru Topirceanu, Mihai Udrescu, Radu Marculescu

**Affiliations:** 1 Department of Computer and Information Technology, Politehnica University of Timişoara, Timişoara, 300223 Romania; 2 Department of Electrical and Computer Engineering, Carnegie Mellon University, Pittsburgh, PA 15213 USA

## Abstract

The dynamics of social networks is a complex process, as there are many factors which contribute to the formation and evolution of social links. While certain real-world properties are captured by the degree-driven preferential attachment model, it still cannot fully explain social network dynamics. Indeed, important properties such as dynamic community formation, link weight evolution, or degree saturation cannot be completely and simultaneously described by state of the art models. In this paper, we explore the distribution of social network parameters and centralities and argue that node degree is not the main attractor of new social links. Consequently, as node betweenness proves to be paramount to attracting new links – as well as strengthening existing links –, we propose the new Weighted Betweenness Preferential Attachment (WBPA) model, which renders quantitatively robust results on realistic network metrics. Moreover, we support our WBPA model with a socio-psychological interpretation, that offers a deeper understanding of the mechanics behind social network dynamics.

## Introduction

Despite the widespread use of the Gaussian distribution in science and technology, many social, biological, and technological networks are better described by a power-law (Zipf) distribution of nodes degree (the node degree is the number of links incident to a node). The Barabasi-Albert (BA) model, based on the degree-driven preferential attachment, generates such scale free networks with a power-law distribution of node degree *P*(*k*) = *k*^−*λ*^. In fact, degree preferential attachment (DPA) is widely considered to be one of the main factors behind complex network evolution (the scale-free topologies generated with the BA model are able to capture other real-world social network properties such as a low average path length *L*)^1,2^. However, recent research challenges the idea that the scale free property is prevalent in complex networks^3^. Additionally, the degree-driven preferential attachment model has well-known limitations to accurately describe social networks (*i.e*., complex networks where nodes represent individuals or social agents, and links represent social ties or social relationships), owing to the following considerations:

- People are physically and psychologically limited to a maximum number of real-world friendships; this imposes a saturation limit on node degree^4,5^. Conversely, in the BA model no such limit exists.
- People have weighted relationships, *i.e*., not all ties are equally important: an average person knows roughly 350 persons, can actively befriend no more than 150 people (Dunbar’s number)^4^, and has only a few very strong social ties (links)^6^. The BA model does not account for such link weights^7^.
- The structure and dynamics of communities in social networks are not accurately described with DPA^7–11^.

To address these issues, recent research has combined the DPA model with properties derived directly from empirical data. For instance, there exist proposals which add the small-world property to scale-free models (*e.g*., Holme-Kim model^12^, evolving scale-free networks^13^) or the power-law distribution to small-worlds (*e.g*., the Watts-Strogatz model with degree distribution^14^, multistage random growing small-worlds^15^, evolving small-worlds^16^, random connectivity small-worlds^17^). Other research proposals extend Milgram’s experiment^18^, *e.g*., static-geographic^19^ and cellular^20^ models. However, all these models are still not accurate enough when compared against real-world social networks.

To better understand the real-world accuracy problem, we perform a topological analysis on a variety of real-world network datasets and show that *node betweenness* (which expresses the node quality of being “in between” communities) is power-law distributed and–at the same time–correlated with link weight distributions. Our empirical findings align well with previous research in some particular cases^11,21^. Such empirical pieces of evidence suggest that, for social networks, the node degree is *not* the main driver of preferential attachment; therefore other centralities may be better attractors of social ties. We conclude that node betweenness–as opposed to node degree or any other centrality metric–is the key attractor for new social ties.

Consequently, as the main theoretical contribution, we introduce the new Weighted Betweenness Preferential Attachment (WBPA) model, which is a simple yet fundamental mechanism to replicate real-world social networks topologies more accurately than other state-of-the-art models. More precisely, we show that the WBPA model is the first social network model that is able to replicate community structure while it simultaneously: (i) explains how link weights evolve, and (ii) reproduces the natural saturation of degree in hub nodes.

Finally, we further interpret WBPA from a socio-psychological perspective, which may explain why node betweenness is such an important factor behind social network formation and evolution.

## Results

### Centrality statistics

We investigate the distributions of node betweenness on a variety of social network datasets: Facebook users (590 nodes), Google Plus users (638 nodes), weighted co-authorships in network science (1589 nodes), weighted online social network (1899 nodes), weighted Bitcoin web of trust (5881 nodes), unweighted Wikipedia votes (7115 nodes), weighted scientific collaboration network (7343 nodes), unweighted Condensed Matter collaborations (23 K nodes), weighted MathOverflow user interactions (25 K nodes), unweighted HEP citations (28 K nodes), POK social network (29 K nodes), unweighted email interaction (37 K nodes), IMDB actors (48 K nodes), Brightkite OSN users (58 K nodes), Facebook - New Orleans (64 K nodes), respectively Epinions (76 K nodes), Slashdot (82 K nodes) and Timik (364 K nodes) on-line platforms. To improve the robustness of our analysis, we ensure data diversity by considering network datasets with different sizes, weighted and unweighted, and representing various types of social relationships (see *Methods*).

**Figure 1:**
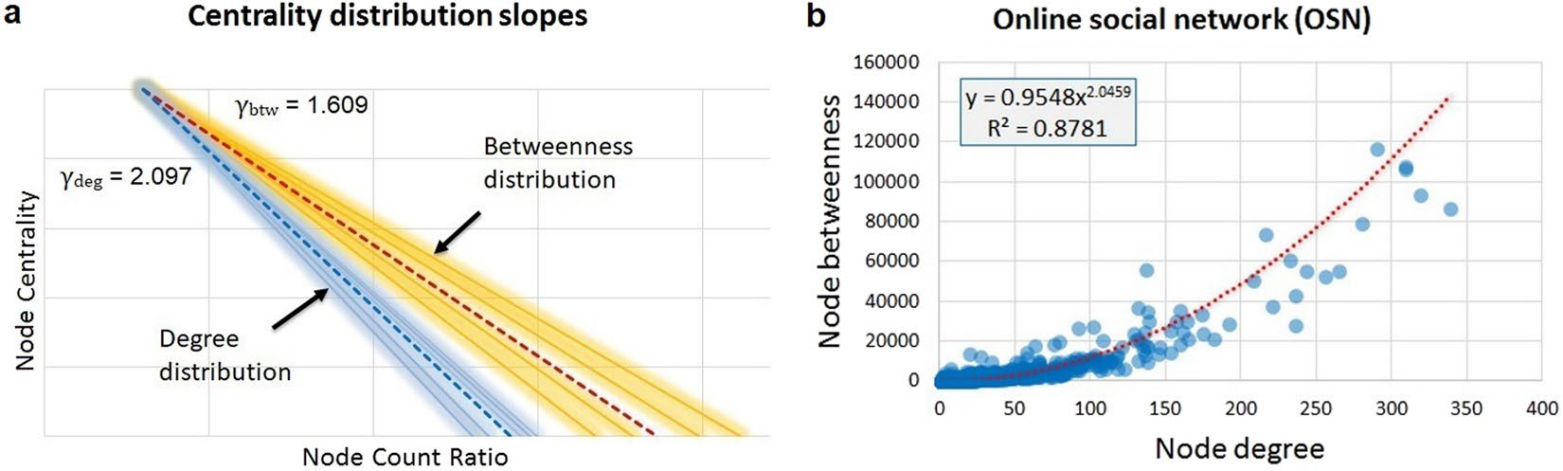
(**a**) Overview of centrality distribution slopes for all empirical datasets; the average slopes are highlighted for node degree (blue) and node betweenness (red). (**b)** Non-linear correlation of node betweenness and node degree in a representative weighted on-line social network (OSN)^22^ with 1899 nodes. These results show that, in social networks, degree and betweenness have a power-law distribution (with a steeper slope for degree), and that there is a non-linear correlation between the two centralities.

Our first observation is that, in all datasets, node degree, node betweenness, link betweenness, and link weights (for datasets with weighted links) are power-law distributed. Moreover, the power-law slope of degree distribution is steeper in comparison with node betweenness distribution. More precisely, as presented in Fig. 1a, the average degree slope is *γ*_*deg*_ = 2.097 (standard deviation *σ* = 0.774) and the average betweenness slope is *γ*_*btw*_ = 1.609 (*σ* = 0.431), meaning that *γ*_*deg*_ is typically 30.3% steeper than *γ*_*btw*_ across all datasets (details in *SI.1. Social network datasets statistics*). Also, for all considered datasets there is a significant non-linear (polynomial or exponential) correlation between node betweenness and node degree (see Fig. 1b); this further suggests that node betweenness may be the source of imbalance in node degree distribution. The statistics for the entire dataset collection are presented in *SI.1*.

**Figure 2:**
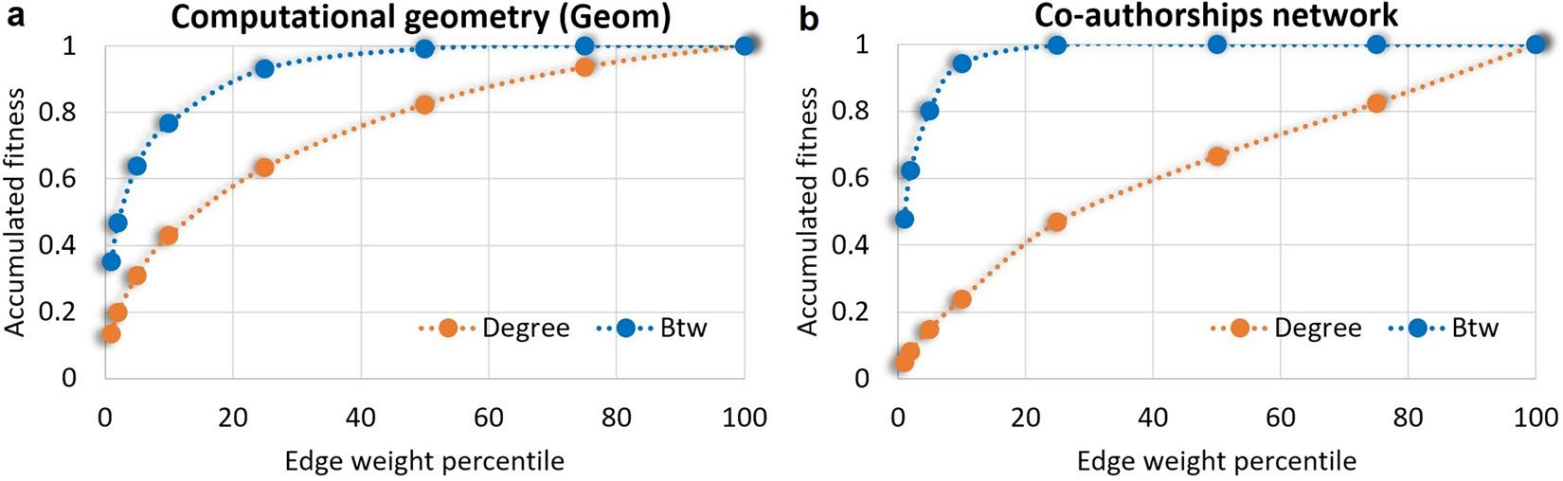
The accumulated fitness (expressed as Degree *D* and Betweenness *B* centralities) of nodes incident to links with weights within the top 1% to 100% percentiles (**a**) in the *Geom* network (7343 nodes, 11898 links), and (**b**) in the *Co-authorships* network (1589 nodes, 2742 links). The Betweenness/Degree ratios (*B*/*D*) range between 2.5–9, highlighting that top link weights are predominantly incident to high betweenness nodes, rather than high degree nodes.

The second observation is that–unlike node degree–node betweenness is significantly more correlated with the weights of the incident links. After assessing the correlation between both node betweenness and node degree with the weighted sum of all adjacent links, we argue that betweenness acts as an attractor for stronger ties. For example, for the co-authorships weighted network with 1589 nodes^23^, the top 5% links accumulate 27.4% of the total weight in the graph; these top 5% links are incident to nodes which amass 80.2% of the total node betweenness, but only 14.9% of the total node degree (see Fig. 2–further numerical details in *SI.1*, Table 2). In all analyzed weighted datasets, node betweenness correlates with incident link weights by ratios that are 2.5–9 times higher than node degree–link weights associations (additional details in *SI.1,* Fig. 2).

**Figure 3:**
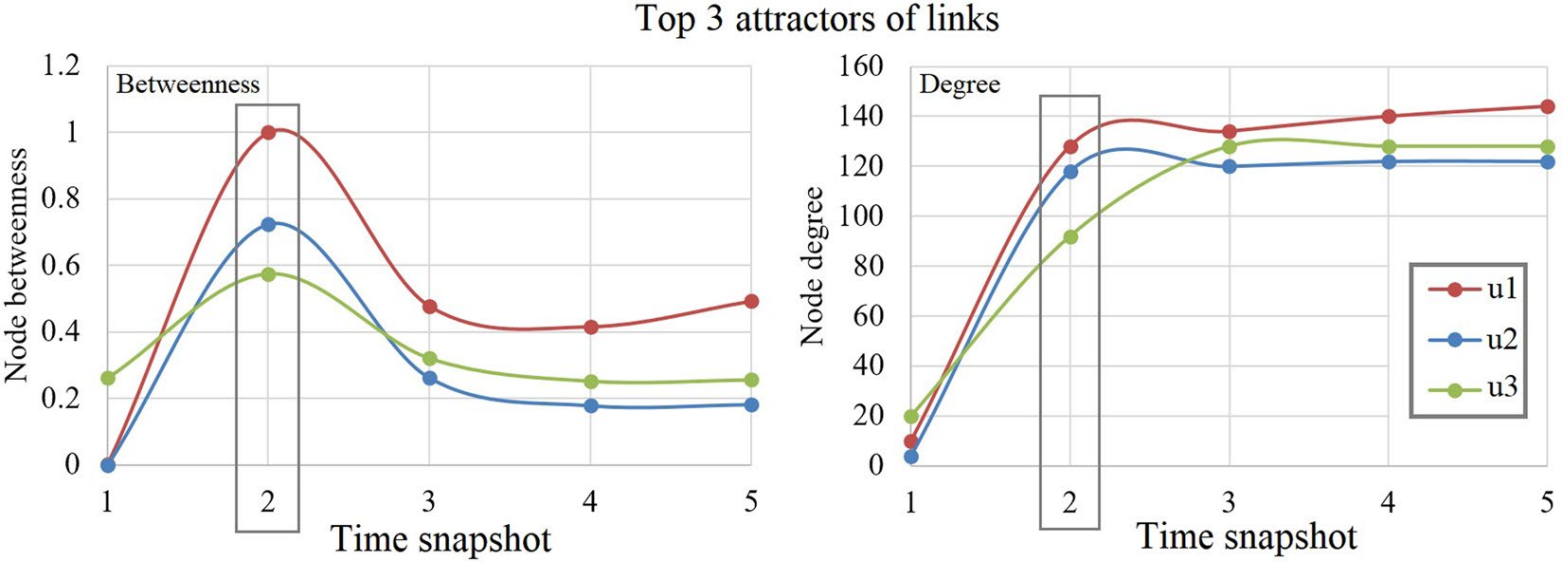
Betweenness and degree evolution for the top 3 link-receiver nodes over time snapshots *T*_1_ − *T*_5_, *i.e*., weeks 2–6 after launching the *UPT.social* network. The three highlighted nodes (anonymized users – *u1, u2, u3*) are the top 3 link receivers at *T*_2_.

The first observation indicates a significant correlation between node degree and node betweenness but it does not necessarily imply causation. However, the second observation is that betweenness attracts stronger links which, in turn, triggers more imbalance in degree distribution; this suggests that *node betweenness* is behind networks evolution, while the power-law degree distribution is only a by-product. The importance of node betweenness is further supported by the analysis of centrality dynamics. To this end, we provide the example of an on-line social network, *UPT.social*, which was intended to facilitate social interaction between students and members of faculty at University Politehnica of Timişoara, Romania^24^. Right after its launch in 2016, *UPT.social* attracted hundreds of users, and the entire dynamical process of new links formation was recorded as snapshots of the first 6 weeks (*T*_0_ − *T*_5_). As exemplified in Fig. 3 (and further detailed in *SI.3*, Fig. 6), the nodes with high betweenness become the principal attractors of new social ties; we also note that the top 3 nodes attracting new edges at time snapshot *T*_2_ are the ones which maximize their betweenness beforehand, and then trigger a subsequent degree increase. As shown, once node degree begins to saturate (*T*_3_ − *T*_5_), node betweenness drops, as nodes fulfill their initial bridging potential.

### Betweenness preferential attachment (BPA)

**Figure 4:**
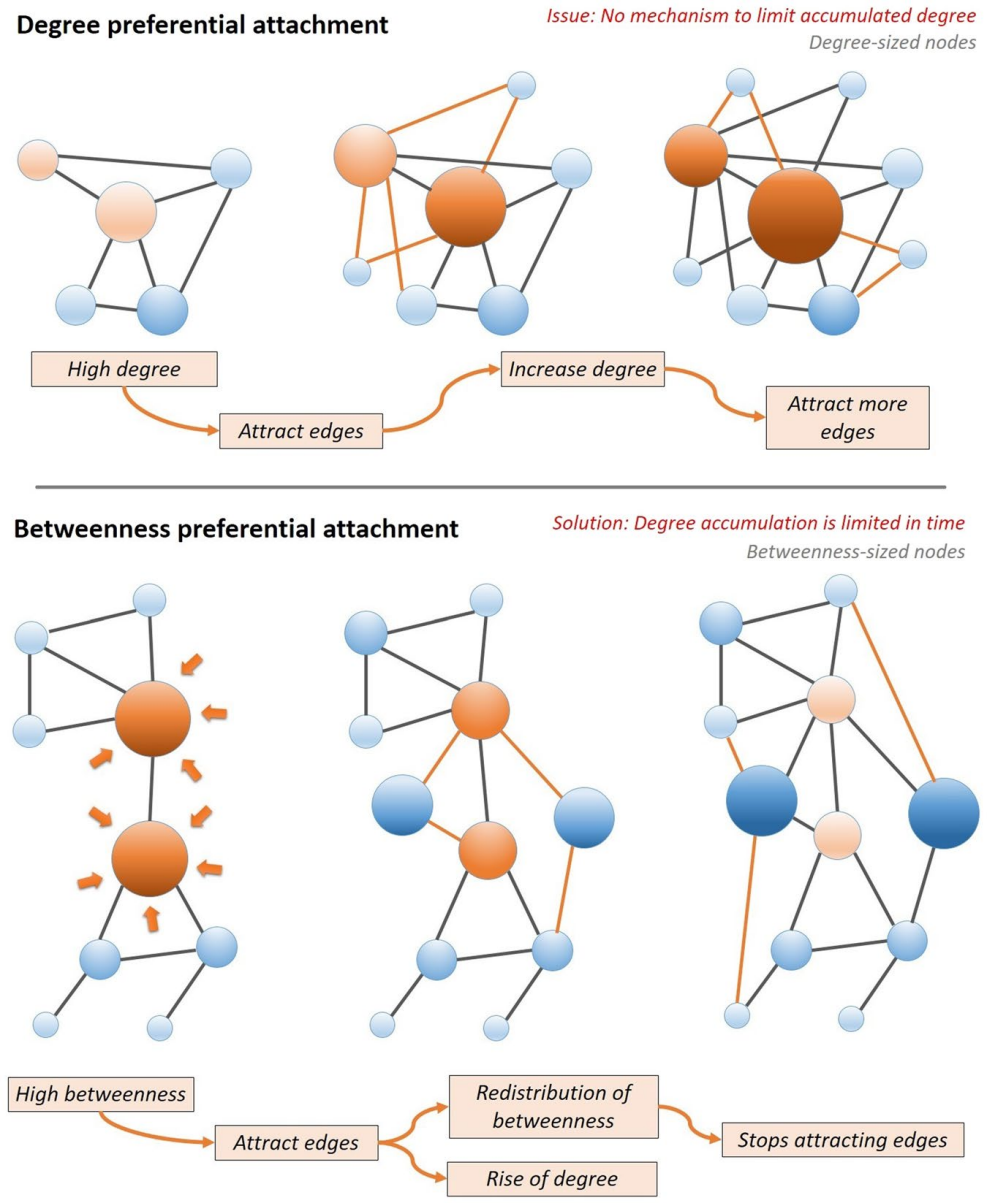
The mechanisms of degree preferential attachment (DPA) versus betweenness preferential attachment (BPA) depicted in terms of acquiring new links and limiting the (excessive) accumulation of degree over time. In DPA, nodes with high degree attract even more links, and thus node degree increases *ad infinitum*. Conversely, in BPA, nodes attracting new links because of their high betweenness will eventually lose their betweenness in favor of their neighboring nodes, thus limiting the acquired degree.

In what follows, we propose the betweenness preferential attachment model (BPA) and conjecture that–for social networks–it is more realistic than the degree preferential attachment (DPA) model. The fundamental difference between the degree-driven and betweenness-driven preferential attachment is illustrated in Fig. 4; the upper panel shows that, under the DPA rule, the nodes with high degree (colored in orange) gain an even higher degree. In contrast, the lower panel in Fig. 4 shows that, under the BPA rule, the nodes with high betweenness (orange) attract more links and increase their degrees; in turn this decreases their betweenness via a redistribution process, thus limiting the number of new links for high-degree nodes as a second order effect. This may explain why, in real-world networks, the number of new links is limited for high degree nodes (*i.e*., degree saturation).

#### WBPA model

Besides validating the BPA mechanism, we also realize that all the empirical network data gathered in a real-world context is *weighted*, even if the information about link weights is not always available. For example, there is no link weight information in our Facebook and Google Plus datasets, yet these networks are clearly part of a weighted social context in which each link has a distinct social strength. Realistic networks evolve according to a mechanism which considers link weights, therefore we develop the weighted BPA (WBPA) algorithm to characterize the social network evolution.

**Figure 5:**
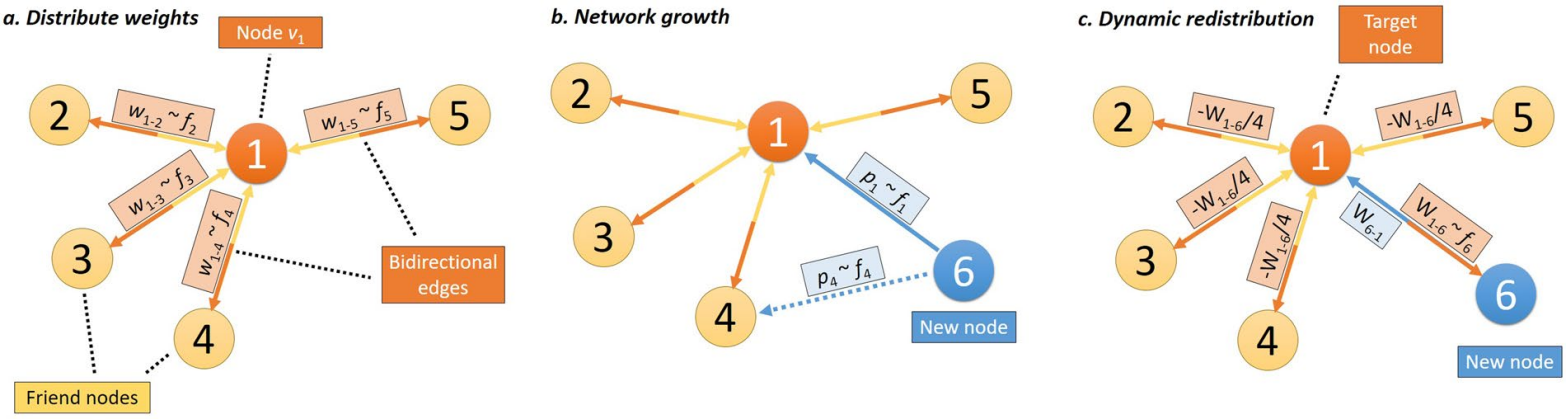
Network evolution according to the Weighted BPA algorithm. (**a)** All bidirectional links *E* in graph *G* are initialized with weights *w*_*ij*_ and *w*_*ji*_, respectively. Each outgoing link weight of node *v*_1_ is proportional to the fitness function (indicated as $w\sim f$) of the target neighbor nodes, and then normalized such that the sum of outgoing weights is 1. (**b)** New node *v*_6_ connects to existing ones *v*_1_–*v*_5_ based on probabilities that are proportional to the normalized fitness ($p\sim f$) of the target nodes. Say, *v*_6_ connects only to *v*_1_ based on fitness *f*_1_. (**c)** Once *v*_6_ and *v*_1_ connect, node *v*_1_ assigns a weight *w*_1−6_ on the new link that is proportional to fitness *f*_6_. As such, a proportional weight ratio of *w*_1−6_/4 is subtracted (indicated with a minus sign) from the four already existing links. If any of the newly resulting weights drop below 0, the corresponding link is removed from node *v*_1_. According to the BPA principle, the fitness *f* is represented by the node betweenness centrality.

The WBPA algorithm for link weight assignment according to the fitness-weight correlation is given in Fig. 5 and discussed below. In the case of WBPA, the fitness *f* is node betweenness. Note that even though link weights *w*_*ij*_ are not used directly during the growth phase, they have a significant second order impact: Betweenness depends on the shortest paths in the graph, which in turn are highly dependent on link weights. Link weights are updated in step 3 of the WBPA algorithm, and whenever a weight becomes ≤0, the corresponding link is removed.

#### Weighted BPA Algorithm (WBPA)

1. ***Distribute weights***: Begin with an arbitrarily connected graph *G* with nodes *V* and bidirectional links *E* (*i.e*., for ∀*e*_*ij*_ ∃ *e*_*ji*_). A weight *w*_*ij*_ is added for each link *e*_*ij*_ in the graph, so that *w*_*ij*_ is proportional to fitness *f*_*j*_ of the target node *v*_*j*_. For each node *v*_*i*_, all incident link weights *w*_*ij*_ are normalized so that the outgoing weighted degree is 1.
2. ***Growth (BPA):*** At every step, a new node *v*_*k*_ is introduced; the new node tries to connect to *n* (1 ≤ *n* ≤ *V*) existing nodes in *G*. The probability *p*_*i*_ that *v*_*k*_ becomes connected to an existing node *v*_*i*_ is proportional to fitness *f*_*i*_. Therefore, we have ${p}_{i}={f}_{i}/{\sum }_{j\in V}\,{f}_{j}$ where the sum is made over all nodes in the graph.
3. ***Dynamic weight redistribution:*** Once a new node *v*_*k*_ becomes connected to an existing node *v*_*i*_, weights *w*_*ki*_ and *w*_*ik*_ are initialized with the normalized fitnesses *f*_*i*_ and *f*_*k*_ respectively. As the weighted outgoing degree of node *v*_*i*_ increases by *w*_*ik*_, every other weight *w*_*ij*_ is rescaled with −*w*_*ik*_/*n*, where *n* is the previous number of neighbors of node *v*_*i*_.

### Assessing the realism of WBPA

WBPA defines complex interactions between link weights and node centralities, hence we expect emerging phenomena such as *n*-order effects. Therefore, a mathematical analysis of WBPA would be cumbersome and beyond the scope of our paper. Instead, as validation strategy, we test WBPA against several preferential attachment (PA) models to explore which one produces the most realistic social network topology. To this end, we quantify preferential attachment according to a fitness function *f* which expresses the capability of individual nodes to attract new connections (*e.g*., if *f* is chosen to be node degree *Deg*, then we reproduce the classic BA model^2^). We consider *f* as one of the following network centralities: degree *Deg* (DPA model), betweenness *Btw* (WBPA model), eigenvector centrality *EC* (ECPA model), closeness *Cls* (ClsPA model), and clustering coefficient *CC* (CCPA model). Each node centrality is defined in the *Methods* section. The comparison between synthetic and real-world networks is done through topological similarity assessment supported by the statistical fidelity metric^25^, alongside standard deviation and p-values. Fidelity takes values *φ* ∈ [0, 1] with 1 representing a network that is identical with the reference network (see the *Methods* section for more details).

**Figure 6:**
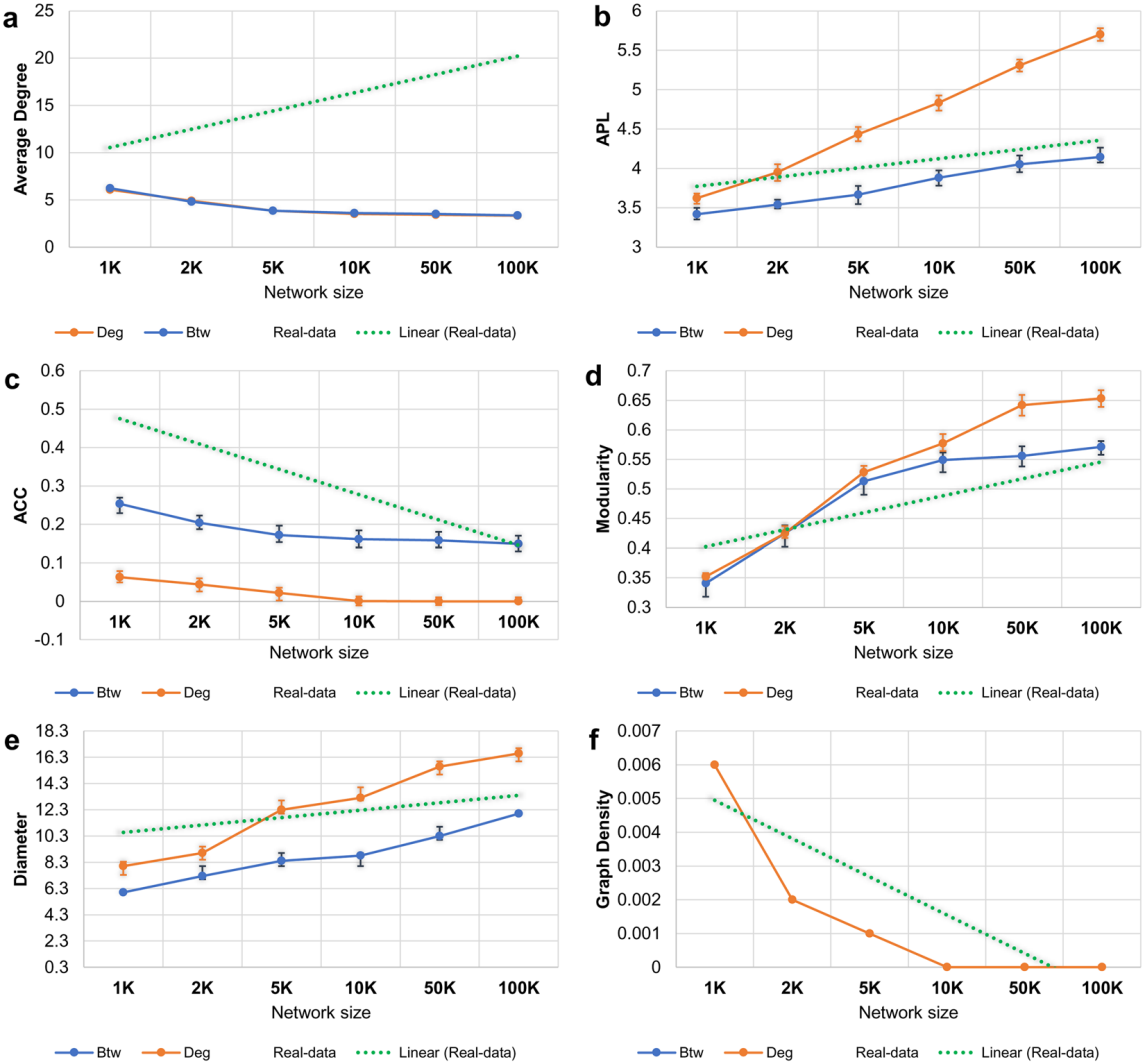
Distribution of the six fundamental graph metrics (**a**–**f**) for increasing networks sizes (*N* = 1 *K* to *N* = 100 *K* nodes) for the real world datasets (green), and the synthetic Preferential Attachment (PA) networks driven by *Btw* (blue) and *Deg* (red). The min-max intervals for each set of measurements are marked with error bars.

We also make use of the following graph metrics to characterize and compare networks: average degree (*AD*), average path length (*APL*), average clustering coefficient (*ACC*), modularity (*Mod*), graph diameter (*Dmt*), and graph density (*Dns*). We start by measuring the distributions of these six metrics on the 18 selected real-word datasets. To assess which centrality is the most appropriate as fitness function, we start by generating networks according to each PA model, of increasing sizes: *N* = {1*K*, 2*K*, 5*K*, 10*K*, 50*K*, 100*K*} nodes; the full statistical results are presented in *SI.2. Best fitness for preferential attachment*. Aggregating the statistical results from *SI.2*–Fig. 4 (real-world data) and Fig. 5 (PA networks), we provide an intuitive visual comparison in Fig. 6 between the *averaged* evolution of the six graph metrics on the real-world data (*N* = 590 to *N* = 364 K nodes), and on the degree-driven and betweenness-driven PA networks.

To better illustrate the comparisons between the synthetic PA networks and the real-world datasets, we present the trend lines for each graph metric in Fig. 6; for the real-world data networks the trend line is green-dotted, for *Btw* fitness networks is blue, and for *Deg* fitness networks is red. On close inspection, we uncover the following:

- *AD* in real data evolves differently than in PA networks.
- *APL* evolution in real data resembles *Btw* networks much better than *Deg* networks. We measure a statistical fidelity of *φ*_*Btw*_ = 0.925 and *φ*_*Deg*_ = 0.853.
- *ACC* evolution in real data resembles *Btw* more than *Deg*, with statistical fidelities of *φ*_*Btw*_ = 0.665 and *φ*_*Deg*_ = 0.515.
- *Mod* evolution in real data resembles both networks very well, with statistical fidelities of *φ*_*Btw*_ = 0.814 and *φ*_*Deg*_ = 0.812 (a slight advantage for the *Btw* networks).
- *Dmt* evolution in real data resembles *Deg* more than *Btw*. Even though we see the same type of increase, *Deg* produces longer diameters as seen in the majority of real-word data. The measured statistical fidelities are *φ*_*Btw*_ = 0.796 and *φ*_*Deg*_ = 0.836.
- *Dns* evolution in real data resembles both networks, with statistical fidelities of *φ*_*Btw*_ = 0.634 and *φ*_*Deg*_ = 0.634.

For simplicity, Fig. 6 includes only *Deg* and *Btw* PA networks in the comparison with real-world data; the full numerical data–with all PA network models–are detailed in Table 1. All these results demonstrate the superior realism provided by the WBPA in comparison to the classic DPA principle, as well as in comparison to PA driven by other node centralities such as eigenvector, closeness or clustering coefficient.

**Table 1:** P-values and fidelity *φ* of WBPA, other PA networks, and the null model (random network) obtained by comparing each individual graph metric with the expected average metrics of the real world datasets.

Metric	WBPA		DPA		ECPA		ClsPA		CCPA		Null	
	*φ*	p-val	*φ*	p-val	*φ*	p-val	*φ*	p-val	*φ*	p-val	*φ*	p-val
*AD*	**0.605**	9.4E-10	0.604	8.4E-10	0.603	9-E10	**0.605**	1E-09	0.603	9E-10	0.603	8E-10
*APL*	**0.925**	0.951	0.853	0.882	0.867	0.972	0.630	0.058	0.665	0.991	0.842	0.882
*ACC*	**0.665**	0.899	0.515	0.872	0.519	0.899	0.503	0.875	0.505	0.879	0.502	0.872
*Mod*	**0.814**	0.998	0.812	0.999	0.812	0.998	0.729	0.982	0.811	0.998	0.798	0.999
*Dmt*	0.796	0.652	**0.836**	0.175	0.821	0.505	0.734	0.017	0.717	0.034	0.795	0.175
*Dns*	0.634	—	0.634	—	0.634	—	**0.685**	—	0.634	—	0.634	—

Bold values represent the highest fidelity on each row *(i.e*., for each graph metric), showing that WBPA obtains the most realistic values for the majority of parameters.

We strengthen our analysis by presenting several direct comparisons between real networks and synthetic PA networks, generated with the same node sizes as the real-world reference networks. The comparisons are made using the fidelity metric *φ*, as well as by comparing individual graph metrics (one by one), to show that WBPA is superior to the other PA networks. To this end, we select the Facebook (FB), Google Plus (GP), Online social network (OSN), and IMDB real-world datasets, and provide the full statistical results in Table 2; here, each sub-table contains the reference real-world network and its graph metrics on the first row, while the remaining lines contain the averaged graph metrics for 10 synthetic networks generated according to preferential attachment driven by each centrality (*Deg*, *Btw*, *EC*, *Cls*, *CC*). Additionally, we provide measurements for a *Null* model (Random network) to serve as baseline. The standard deviation for each synthetic dataset metric is symbolized with a ± sign.

**Table 2:** Topological comparison of the Facebook (FB), Google Plus (GP), Online social network (OSN), and actors’ IMDB datasets with the five preferential attachment network models, and a baseline random network (null model).

Datasets	AD	APL	ACC	Mod	Dmt	Dns	*φ*	p-val
FB	19.82	2.481	0.266	0.468	8	0.005		
Null	9.986 ± 0.165	2.448 ± 0.014	0.034 ± 0.001	0.229 ± 0.001	4 ± 0.000	0.054 ± 0.000	0.598	0.1936
DPA	8.755 ± 0.097	4.526 ± 0.220	0.018 ± 0.067	0.615 ± 0.003	9.7 ± 0.006	**0.005** ± **0.577**	0.731	0.1313
WBPA	8.908 ± 0.154	**3.142** ± **0.101**	**0.259** ± **0.021**	**0.568** ± **0.020**	**6** ± **0.000**	**0.005** ± **0.000**	**0.878**	0.2435
ECPA	8.906 ± 0.178	4.318 ± 0.030	0.016 ± 0.002	0.616 ± 0.002	9.5 ± 0.707	**0.005** ± **0.000**	0.738	0.1599
ClsPA	8.812 ± 0.103	5.764 ± 0.167	0.006 ± 0.001	0.634 ± 0.019	13.5 ± 2.121	**0.005** ± **0.000**	0.653	0.0124
CCPA	8.934 ± 0.114	3.924 ± 0.107	0.007 ± 0.002	0.622 ± 0.004	7 ± 1.000	**0.005** ± **0.001**	0.756	0.2029
**Datasets**	**AD**	**APL**	**ACC**	**Mod**	**Dmt**	**Dns**	***φ***	**p-val**
**GP**	**12.15**	**3.9**	**0.404**	**0.44**	**12**	**0.035**		
Null	12.129 ± 0.109	2.344 ± 0.006	0.038 ± 0.001	0.203 ± 0.002	3 ± 0.000	**0.038** ± **0.000**	0.676	0.1658
DPA	8.351 ± 0.156	2.664 ± 0.001	0.051 ± 0.003	0.238 ± 0.001	4.5 ± 0.707	0.021 ± 0.000	0.662	0.2460
WBPA	8.370 ± 0.139	2.384 ± 0.007	**0.269** ± **0.027**	**0.275** ± **0.006**	5 ± 0.000	0.021 ± 0.000	**0.709**	0.3100
ECPA	8.614 ± 0.113	2.683 ± 0.018	0.042 ± 0.003	0.232 ± 0.006	4.4 ± 0.548	0.021 ± 0.000	0.659	0.2479
ClsPA	8.242 ± 0.112	2.770 ± 0.150	0.027 ± 0.002	0.232 ± 0.018	5 ± 1.000	0.021 ± 0.000	0.664	0.2944
CCPA	8.262 ± 0.219	**2.773** ± **0.090**	0.028 ± 0.002	0.247 ± 0.006	**6.3** ± **0.577**	0.021 ± 0.000	0.676	0.4553
**Datasets**	**AD**	**APL**	**ACC**	**Mod**	**Dmt**	**Dns**	***φ***	**p-val**
**OSN**	**10.68**	**3.055**	**0.138**	**0.249**	**8**	**0.008**		
Null	10.807 ± 0.121	2.777 ± 0.008	0.011 ± 0.001	0.211 ± 0.002	4.3 ± 0.577	0.012 ± 0.000	0.731	0.8718
DPA	11.789 ± 0.126	3.078 ± 0.018	0.019 ± 0.003	0.232 ± 0.008	**8.6** ± **0.547**	0.006 ± 0.000	0.839	0.9973
WBPA	11.911 ± 0.163	**3.035** ± **0.005**	**0.242** ± **0.023**	**0.252** ± **0.007**	**7.4** ± **0.894**	**0.007** ± **0.001**	**0.874**	0.9982
ECPA	11.793 ± 0.101	**3.075** ± **0.210**	0.021 ± 0.004	0.253 ± 0.006	7 ± 1.000	0.006 ± 0.000	0.842	0.9968
ClsPA	11.807 ± 0.103	3.319 ± 0.159	0.009 ± 0.002	0.263 ± 0.022	12.7 ± 0.57	0.007 ± 0.001	0.781	0.7022
CCPA	11.681 ± 0.224	3.155 ± 0.007	0.006 ± 0.001	0.221 ± 0.010	6.4 ± 0.547	0.006 ± 0.000	0.802	0.9903
**Datasets**	**AD**	**APL**	**ACC**	**Mod**	**Dmt**	**Dns**	***φ***	**p-val**
**IMDB**	**23.02**	**3.772**	**0.197**	**0.63**	**13**	**0.001**		
Null	3.396 ± 0.134	5.501 ± 0.010	1E-4 ± 0.000	0.49 ± 0.002	14.3 ± 0.577	1E-6 ± 0.000	0.682	0.0031
DPA	3.438 ± 0.101	5.288 ± 0.002	1E-4 ± 0.000	0.642 ± 0.009	15.5 ± 0.600	**1E-4** ± **0.000**	0.712	0.0030
WBPA	**3.526** ± **0.040**	**4.045** ± **0.035**	**0.159** ± **0.021**	0.555 ± 0.018	10.2 ± 0.690	**1E-4** ± **0.000**	**0.803**	0.0043
ECPA	3.461 ± 0.111	5.103 ± 0.019	0.006 ± 0.001	**0.634** ± **0.005**	**12.0** ± **0.700**	**1E-4** ± **0.000**	0.739	0.0039
ClsPA	3.475 ± 0.077	7.611 ± 0.119	1E-4 ± 0.000	0.741 ± 0.016	18.8 ± 0.400	**1E-4** ± **0.000**	0.613	0.0003
CCPA	3.413 ± 0.134	4.862 ± 0.004	1E-4 ± 0.000	0.621 ± 0.006	8.0 ± 0.333	**1E-4** ± **0.000**	0.702	0.0018

Standard deviation is marked with ±. Bold values on each column represent the closest match to the reference network. A higher fidelity *φ* means a closer match with the reference network.

The mechanism of preferential attachment which we adopt in our paper is a fundamental, yet generic and simple framework. State of the art studies which are specifically aimed at creating realistic topologies propose algorithms with a far increased complexity. Therefore, intuitively, it is expected that state of the art models like Cellular (Cell)^20^, Home-Kim (HK)^12^, Toivonen (TV)^26^, or Watts-Strogatz with degree distribution (WSDD)^14^ etc., will generate more realistic topologies in terms of the six discussed graph metrics. To test this hypothesis, we further generate such synthetic networks of size *N* = 10,000 and compare them with WBPA, DPA networks and several real-world datasets. The results are provided in Table 3, showing that not only is WBPA superior to DPA and PA models driven by other centralities but, in most cases (*i.e*., 10 out of 13), it outperforms the other synthetic models in terms of topological fidelity as well. For readability purposes we did not add information about the standard deviations of each synthetic model here; this information may be found in *SI.4*, Tables 4 and 5.

**Table 3:** Statistical fidelity *φ* of WPBA, DPA, two *Null* models (random and small-world), and four state of the art network (Cellular, Holme-Kim, Toivonen, Watts-Strogatz with degree distribution) models, obtained by comparing the topologies with multiple real-world datasets.

Datasets	*φ*_*FB*_	*φ*_*GP*_	*φ*_*CoAu*_	*φ*_*OSN*_	*φ*_*BTC*_	*φ*_*MOvr*_	*φ*_*HEP*_	*φ*_*POK*_	*φ*_*EmE*_	*φ*_*IMDB*_	*φ*_*BK*_	*φ*_*FBNO*_	*φ*_*TK*_
WBPA	**0.835**	**0.842**	0.735	**0.801**	**0.897**	**0.814**	**0.845**	**0.771**	0.837	**0.892**	0.779	**0.888**	**0.871**
DPA	0.694	0.796	**0.778**	0.634	0.754	0.692	0.836	0.758	**0.851**	0.838	0.782	0.849	0.839
Rand	0.681	0.719	0.681	0.597	0.816	0.761	0.779	0.754	0.733	0.774	0.678	0.788	0.753
SW	0.737	0.718	0.705	0.554	0.644	0.579	0.603	0.669	0.769	0.643	**0.824**	0.612	0.657
Cell	0.543	0.707	0.637	0.52	0.566	0.559	0.503	0.508	0.792	0.55	0.622	0.501	0.591
HK	0.704	0.778	0.578	0.66	0.687	0.679	0.522	0.577	0.787	0.579	0.648	0.539	0.675
Tvn	0.638	0.676	0.711	0.55	0.571	0.561	0.558	0.601	0.831	0.569	0.676	0.56	0.612
WSDD	0.497	0.708	0.673	0.443	0.547	0.535	0.511	0.556	0.825	0.516	0.627	0.513	0.591

Values in bold represent the highest fidelity on each column (*i.e*., most realistic topology).

To offer the diversity required by a robust test of our model, we also include unweighted networks in our collection. A fair comparison between WBPA networks (which are all weighted) and the large and unweighted example networks, requires that all weights on our WBPA algorithm output be discarded. In this comparison, we start by generating WBPA networks of 10,000 nodes, then make all weights ${w}_{ji} > 0$ become 1, thus obtaining unweighted BPA networks.

The upper half of Table 3 contains the average fidelities of WBPA, DPA and the two null model networks, towards the real-world reference networks. The lower half of Table 3 contains the other state of the art synthetic networks. Our WBPA obtains the highest fidelity towards most empirical references, *e.g*., 13–68% higher *φ*_*FB*_, 21–81% higher *φ*_*OSN*_, 4–47% higher *φ*_*TK*_ than all other synthetic models. As such, we prove the increased realism of our model in comparison with some elaborated state-of-the-art models (briefly described in *SI.4*, and quantified in *SI.4*, Table 4). Compared to DPA, our model produces networks with higher fidelity values; when averaged over all empirical networks we obtain: ${\overline{\varphi }}_{Btw}=0.831$ and ${\overline{\varphi }}_{Deg}=0.777$.

We note that the WBPA model produces a specific distribution of the Betweenness/Degree (B/D) ratio. To this end, we measure B/D distributions on all datasets (weighted and unweighted), as well as on our synthetic WBPA-generated networks, using the Gini coefficient (a Gini coefficient takes values between 0 and 1, with values closer to 0 representing a more uniform dispersion of data) to evaluate data dispersion^27^. The Gini values obtained on the empirical data are given in Table 4: all empirical datasets, whether weighted or unweighted, have their Gini coefficients within a similar range, *i.e*., the average real-world Gini is *g*_*real*_ = 0.5193 ± 0.071. Indeed, for WBPA networks with 10,000 nodes, we have an average Gini coefficient of *g*_*WBPA*_ = 0.4962 ± 0.0282, which is very close to the real-world B/D Gini values (−4.5%). Additionally, we generate 10 of each random, small world, and PA networks of 10,000 nodes. For these synthetic networks we obtain the corresponding Gini values in Table 4. The PA networks (except WBPA) produce an average *g*_*PA*_ = 0.7784 ± 0.0128, whereas the random network produces an average Gini *g*_*rand*_ = 0.9374 ± 0.0013. These results point out two key aspects: (i) the B/D dispersion in other PA and other state-of-the-art synthetic models differs significantly from real-world social networks, and (ii) WBPA produces networks with B/D distributions that are closer to the real-world.

**Table 4:** Gini coefficients *g* for the distributions of betweenness/degree (B/D) ratios in real-world networks (ranging between 590–82 K nodes and 2742–948 K links), null-model synthetic networks (random, small-world), and PA networks (10 K nodes).

Datasets	*g*	*σ*	Δ_*real*_	Δ_*real*_%
Facebook	0.5955	—	—	—
Google-Plus	0.4820	—	—	—
Co-authorships	0.4392	—	—	—
Online SN	0.5921	—	—	—
POK	0.4879	—	—	—
Random	0.9374	0.0013	0.418	+80.5%
Small-world	0.8771	0.0451	0.358	+68.9%
DPA	0.7784	0.0182	0.263	+50.7%
ECPA	0.7767	0.0038	0.257	+59.6%
ClsPA	0.7617	0.0017	0.242	+46.7%
CCPA	0.7924	0.0203	0.273	+52.6%
WBPA	0.4962	0.0282	**0.023**	**−4.5%**

For synthetic networks we specify the standard deviation *σ* (after generating 10 networks of each), and the difference towards the empirical average Gini coefficient *g*_*real*_ = 0.5193 (absolute value Δ_*real*_ and relative percentage Δ_*real*_%).

**Figure 7:**
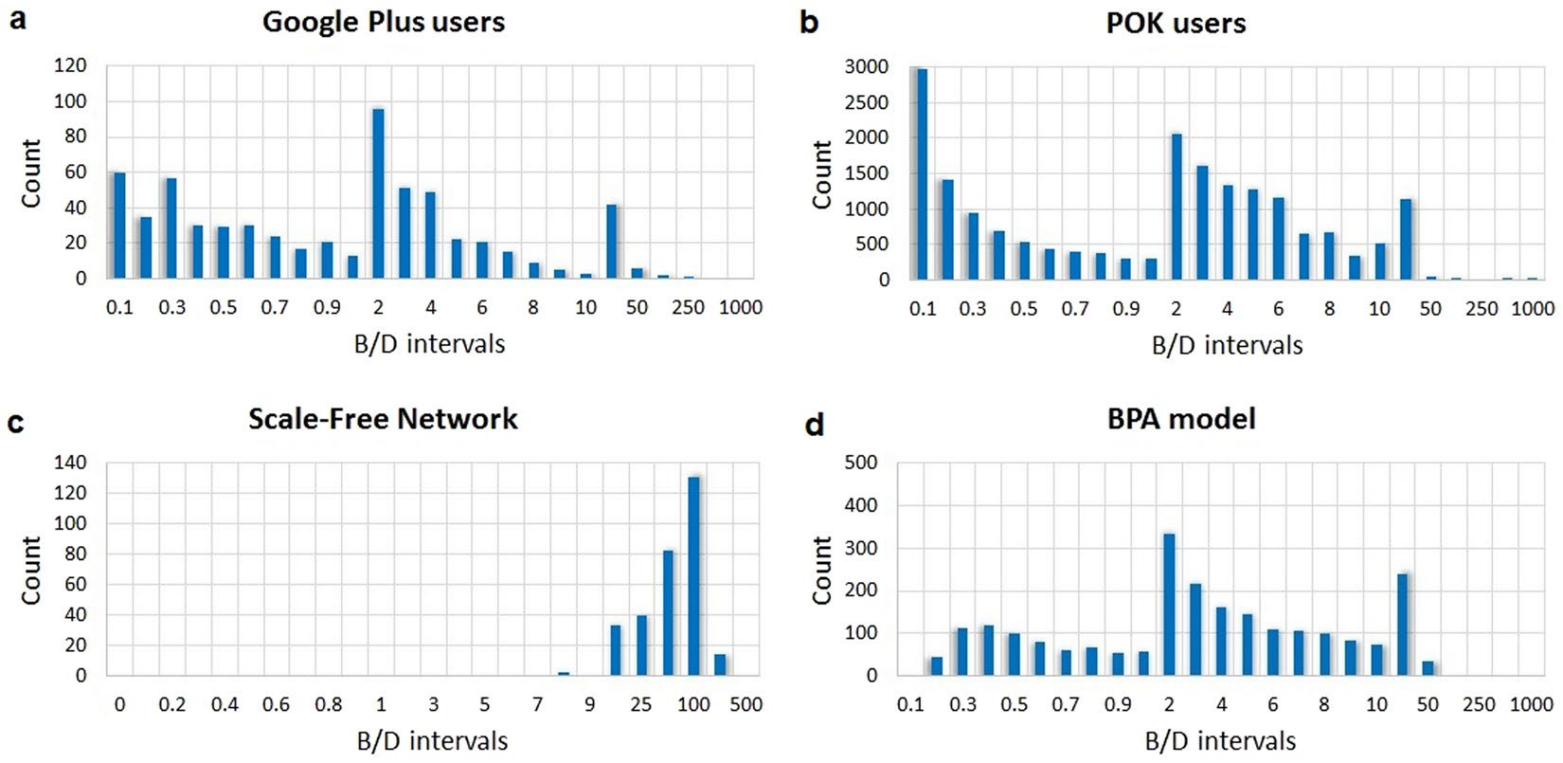
Distributions of betweenness/degree (B/D) ratios in empirical and synthetic social networks characterized by Gini coefficients *g*. (**a**) Google Plus users network^28^ (*g*_*GP*_ = 0.4820). (**b**) POK users network^29^ (*g*_*PK*_ = 0.4879). (**c**) DPA network^2^ (*g*_*DPA*_ = 0.7828 ± 0.0182) (**d**) WBPA network (*g*_*WBPA*_ = 0.4962 ± 0.0282). The B/D distribution in our WBPA network model, as opposed to the DPA network, is very similar to that found in real-world networks.

Two specific B/D distributions are exemplified in Fig. 7a,b for the Google Plus and POK users networks, respectively. Figure 7c,d present the B/D distribution for the DPA and WBPA networks. The visual similarity inspection reveals WBPA as the only synthetic model capable of reproducing the real-world B/D ratios (see *SI.1,* Fig. 3 for additional examples).

The WBPA realism is also backed up by the centrality distribution analysis. The power-law slopes for degree and betweenness distributions in WBPA (*γ*_*deg*_ = 1.391 and *γ*_*btw*_ = 1.171) are very similar to the real-world distributions from the *Centrality statistics* section (see Fig. 1) and *SI.1*, Table 1, meaning that the degree slope is steeper than the betweenness slope (with 18.8%). Similar to the real-world cases, we obtain a polynomial fit for the node betweenness-degree correlation in WBPA (*y* = 0.246*x*^2^ + 329.8*x* − 3569.4, with correlation coefficient *R*^2^ = 0.9977).

## Discussion and a Socio-Psychological Interpretation

From a computational standpoint, node betweenness is significantly more complex to compute in comparison with node degree. However, when individuals make assessments of social attractiveness in real-world situations–which is essential for driving preferential attachment and establishing new social links–they do not rely on executing algorithms or other types of quantitative evaluations. Instead, individuals make decisions based on qualitative perceptions^30^. In light of the *quality over quantity* hypothesis proposed by social psychology^31^, we argue that node betweenness is a far better indicator of social attractiveness than node degree, because the quality of being “in between” can be easily and quickly perceived, due to the fact that humans are better at observing qualitative aspects (*e.g*., differences and diversity) than quantitative ones^32^. This idea is supported by an experimental study on how people favor investing in fewer qualitative social ties, rather than numerous lower quality ties^32^. Our results indicate that WBPA provides a more accurate social network topological model, being able to reproduce real-world community structure as well as to explain degree saturation and link weight evolution.

We believe that the WBPA model transcends the mere topological perspective on social relationships evolution. As such, in the field of social psychology, individuals are perceived as *social creatures* who strive for social recognition, validation, approval and fame^7,19,33,34^. Indeed, individuals tend to connect to two types of other nodes: individuals who are popular in their communities (*i.e*., typically they have high degree), and individuals who connect multiple communities (having high betweenness). While the former type of interconnection is mostly related to the popularity of individuals within local communities, it appears to be an epiphenomenon of the latter.

Also, state of the art has previously identified that social networks have apparent (degree) assortative mixing, while, technological and biological networks appear to be disassortative in nature^34,35^. The study in^35^ explains this as most networks have a tendency to evolve, unless otherwise constrained, towards their maximum entropy state–which is usually disassortative. A similar debate was introduced by Borondo et al. based on the concepts of meritocracy versus topocracy^36^. The authors discuss the critical point at which social value changes from being based on personal merit, to being based on social position, status, and acquaintances. In the context of social networks, we interpret this issue as follows: in our ego-networks the balance between friends with less influence and ones with more influence than us translates into betweenness assortativity. Indeed, connecting to persons with high betweenness and increasing our tie strength with them (through, say, a stable social relationship), we ourselves become, in turn, more influential social bridges. This propagation of influence determines other persons, with lower betweenness, to interact with us and direct more tie strength towards us.

Towards this end, we introduce the concept of *social evolution cycle*, which revolves around betweenness assortativity rather than degree assortativity^34,35,37^. According to our approach, individuals become more influential over time by increasing their own betweenness. Therefore, the exhibition of one individual’s desire to increase his/her betweenness is two-fold: it attracts new ties (*i.e*., increase in degree), and it creates stronger ties (*i.e*., increase in link weight); this process continues for the next generation of individuals who aspire to climb the social ladder. As shown, this conclusion is supported by the evolution of networks generated with WBPA.

**Figure 8:**
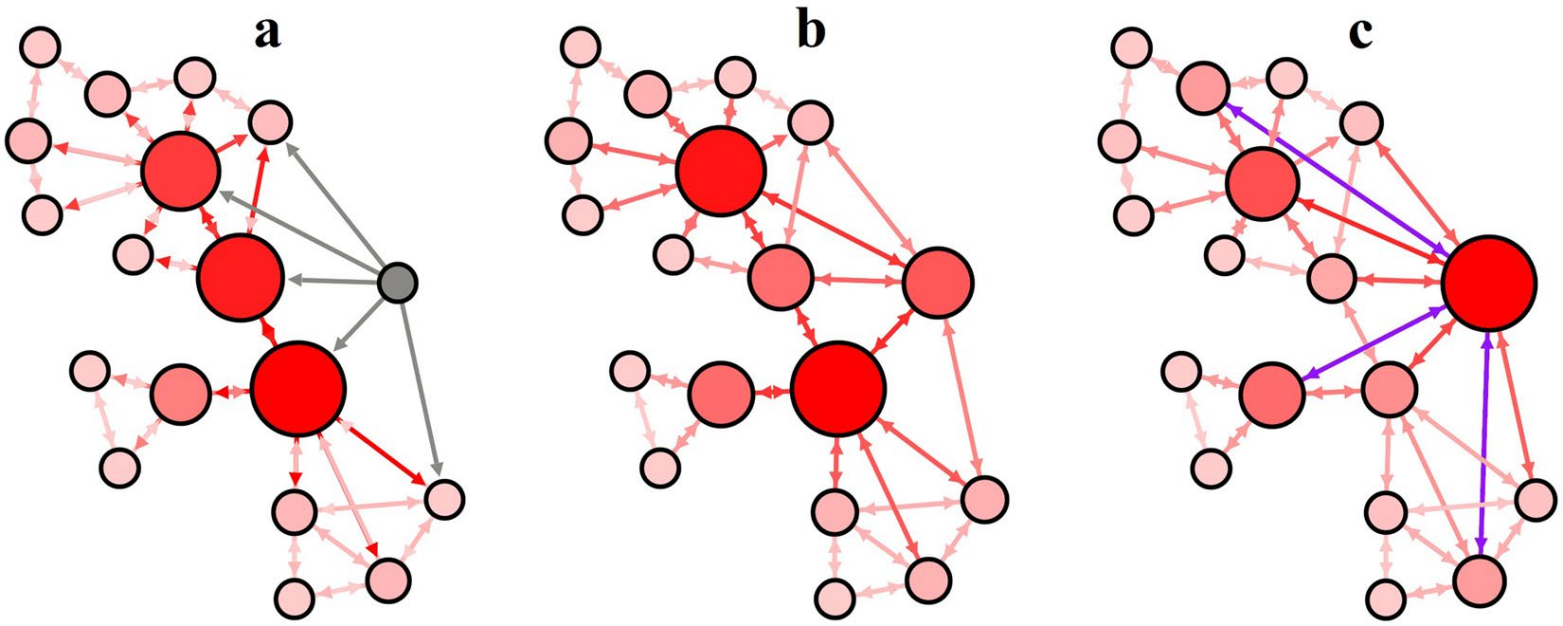
An intuitive explanation of the social evolution cycle. All nodes are colored and sized proportional to their betweenness centrality (influence). (**a**) A non-influential individual (grey) initiates social contact (link) with other individuals equal or more influential than himself. (**b**) This action leads to a natural increase of the individual’s influence (betweenness). (**c**) Other nodes with less influence start connecting to the initial individual. At this point, the initial node has become a predominant receiver of new ties, as emphasized by the new violet links.

We envision two ways of improving an individual’s social status. The first choice relies on forcing tie strengths inside the existing neighborhood to increase first, followed by an increase in influence. The second choice relies on increasing influence first by broadening the neighborhood to influential agents (BPA principle), which will in turn trigger an increase in tie strengths. We consider the second choice as the more plausible social process, as detailed and explained in Fig. 8.

We conclude that the WBPA model is quantitatively more robust than DPA, as it can reproduce more accurately a wide range of real-world social networks. Such a conclusion means that node degree is not the main driver in social network dynamics. Instead, node betweenness is a much better indicator of social attractiveness, because it drives the formation of new social bonds, as well as the evolution of social status of individuals. From a socio-psychological standpoint, individuals (intuitively) perceive node’s betweenness as the capacity of bridging communities, irrespective of its degree. As shown, WBPA is a subtle mechanism at work that is able to replicate the social network community structure. Also, WBPA explains the dynamic accumulation of degree and link weights, as well as the eventual degree saturation, as a second order effect. Consequently, we believe our work paves the way for a new and deeper understanding of the mechanisms that lie behind the dynamics of complex social networks.

## Methods

### Real-world datasets

All data used in this study were selected to facilitate a thorough analysis of node betweenness and degree, as well as measuring the realism of synthetic networks. The real-world datasets have been chosen based on diversity of both context and network size. Prior studies confirm that data mining from sources such as Facebook or Google Plus is reliable for realistic social network research^38,39^, and indicate a strong correlation between the real-world and virtual friendships of people^40,41^.

Table 5 provides the graph metric measurements used for the realism assessment of our WBPA model, as presented in the *Results* section. Our real-world datasets comprise the following social networks (ordered by network size, from *N* = 590 to *N* = 364*K* nodes): Facebook (FB) users^41^, Google Plus (GP) users^28^, weighted co-authorships (CoAu) in network science^23^, weighted on-line social network (OSN)^22^, trade network using Bitcoin OTC platform (BTC)^42^, votes for Wikipedia administrators (WkV)^43^, weighted scientific collaboration network in Computational Geometry (Geom)^44^, Condensed Matter collaboration network from arXiv (CM)^45^, weighted interactions on the stack exchange web site MathOverflow (MOvr)^46^, High-Energy Physics citation network (HEP)^47^, POK online social network^29^, Enron email (EmE) communication network^48^, IMDB adult actors co-appearances, Brightkite online social network (BK)^49^, Facebook-New Orleans (FBNO)^50^, Epinions online social network (EP)^51^, Slashdot online social network (SL)^48^, and Timik online platform (TK)^52^.

**Table 5:** Network sizes (numbers of nodes *N* and edges *E*) and mean values of average degree (*AD*), average path length (*APL*), average clustering coefficient (*ACC*), modularity (*Mod*), diameter (*Dmt*), and density (*Dns*) for the chosen real-world datasets.

Dataset	Acronym	*N*	*E*	*AD*	*APL*	*ACC*	*Mod*	*Dmt*	*Dns*
Facebook	FB	590	5847	19.82	2.481	0.266	0.468	8.5	0.05
Google Plus	GP	638	3875	12.15	3.9	0.404	0.44	12	0.035
Co-authorships	CoAu	1589	2742	3.451	5.823	0.878	0.954	17	0.002
Online social network	OSN	1899	20296	10.68	3.055	0.138	0.249	8	0.008
Bitcoin OTC	BTC	5881	21492	7.309	3.571	0.288	0.489	9	0.001
Wikipedia votes	WkV	7115	101 K	28.32	3.248	0.209	0.421	7	0.004
Geometry collaboration	Geom	7343	11898	3.241	5.313	0.728	0.783	14	0
CondMat collaboration	CM	23 K	93 K	8.083	5.352	0.706	0.729	15	0
MathOverflow	MOvr	25 K	188 K	15.15	3.231	0.412	0.351	9	0.001
HEP citations	HEP	28 K	353 K	25.40	4.278	0.119	0.65	15	0.001
POK social network	POK	29 K	115 K	18.75	5.2	0.109	0.3	11	0
Email Enron	EmE	37 K	184 K	10.02	4.025	0.716	0.618	13	0
IMDB co-appearances	IMDB	48 K	1.1 M	23.02	3.772	0.197	0.63	13	0.001
Brighkite social network	BK	58 K	214 K	7.353	7.371	0.271	0.674	18	0
Facebook New-Orleans	FBNO	64 K	1.5 M	24.25	4.349	0.148	0.61	15	0.001
Epinions social network	EP	76 K	508 K	13.41	4.307	0.066	0.445	14	0
Slashdot social network	SL	82 K	948 K	23.08	4.069	0.024	0.343	11	0
Timik platform	TK	364 K	6.1 M	33.28	4.086	0.117	0.52	14	0

Information about the nature of nodes and links, as well as direct URLs for each dataset are provided in *SI.5 Datasets availability*, Table 6. In the main manuscript, Table 6 presents the natural ranges for the graph metrics that are provided in Table 5, as they are measured across the entire range of considered real-world on-line social networks^41^.

**Table 6:** Natural ranges for considered graph metrics: average degree (*AD*), average path length (*APL*), average clustering coefficient (*ACC*), modularity (*Mod*), diameter (*Dmt*), and density (*Dns*).

Dataset	*AD*	*APL*	*ACC*	*Mod*	*Dmt*	*Dns*
Range	8.57–37.18	1.92–3.04	0.215–0.299	0.313–0.656	6–11	0.02–0.114
Average	20.02	2.48	0.265	0.472	8.41	0.0512
*σ*	7.898	0.239	0.023	0.096	1.19	0.022

### Network centralities

All graphs are generated and visualized using *Gephi*^53^; the graph centralities are analyzed using the *poweRlaw* package distributed with *R* according to the methodology described in^54^. Full details for the topological analysis of data are given in *SI.1*. Furthermore, to quantify the specific distributions of B/D ratios introduced in this paper we made use of the Gini coefficient–borrowed from the area of economics where it is used to evaluate data dispersion^27^.

In *SI.2* we present the preferential attachment analysis based on combinations of two and three node centralities. Given a graph *G* = (*V*, *E*), with nodes *v*_*i*_ ∈ *V* and links *e*_*ij*_ ∈ *E*, we define the basic graph centralities and metrics used throughout the paper. We represent the adjacency matrix as *W* = {*w*_*ij*_}, which contains either the weight of the link for any link *e*_*ij*_, or 0, if no link exists. If the network is unweighted, then each *w*_*ij*_ = 1.

The *degree k*_*i*_ of a node *v*_*i*_ (also denoted as *D*) is defined as ${k}_{i}=\sum {w}_{ij}$. In case of directed networks, there is a differentiation between in-degree and out-degree, but that is beyond the scope of this subsection. The *average degree AD* of the graph is calculated over all nodes as^1^:

1 $$AD=\frac{1}{n}\sum _{i\in G}{k}_{i}$$

The *clustering coefficient CC*_*i*_ measures the fraction of existing links in the vicinity *V*_*i*_ of a node, and is formally defined as^55^:

2 $$C{C}_{i}=\frac{|\{{e}_{jk}\,|\,j,\,k\in {V}_{i}\}|}{{k}_{i}({k}_{i}-\mathrm{1)}}$$

with *k*_*i*_ being the degree of node *v*_*i*_, and *e*_*jk*_ the set of links connecting two friends in the vicinity of node *v*_*i*_, all divided by the maximum number of links in vicinity *V*_*i*_. Consequently, the average clustering coefficient *ACC* of the entire graph is the average of all *CC*_*i*_ over all nodes.

Considering *d*(*v*_*i*_, *v*_*j*_) as the shortest path between two nodes in *G*, the average path length *APL* is defined as^1^:

3 $$APL=\frac{1}{n(n-\mathrm{1)}}\sum _{i\ne j\in G}d({v}_{i},{v}_{j})$$

If there is no path between two nodes, then that particular distance is considered 0; *n* is the total number of nodes |*V*| in *G*.

The *diameter* of a graph is defined as the longest geodesic^56^, namely the longest shortest distance between any two nodes: *Dmt* = *max*(*d*(*v*_*i*_, *v*_*j*_)).

Graph *density* is simply defined as the ratio between number of links and maximum possible number of links, if the graph were complete^56^. For undirected graphs, it is defined as:

4 $$Dns=\frac{\mathrm{2|}E|}{n(n-\mathrm{1)}}$$

*Modularity* is a measure for quantifying the strength of division of a graph into modules, or clusters, and is often used in detection of community structure^57^. Modularity *Mod* is the fraction of the links which lie within a given group minus the expected fraction if links were distributed at random. Values for *Mod* range between [−1/2, 1). If it is positive, then the number of links within a cluster exceeds the expected number. Also, a high overall modularity means dense connections between the nodes within modules and sparse connections between nodes in different modules. We use the algorithm of Blondel et al. to compute modularity^58^.

*Betweenness* centrality is commonly defined as the fraction of shortest paths between all node pairs that pass through a node of interest^1^, and is defined as^59^:

5 $$Btw({v}_{i})=\sum _{i\ne j\ne k\in G}\frac{{\sigma }_{jk}({v}_{i})}{{\sigma }_{jk}}$$

where *σ*_*jk*_(*v*_*i*_) is the number of shortest paths in *G* which pass through node *v*_*i*_, and *σ*_*jk*_ is the total number of shortest paths between all pairs of two nodes *v*_*j*_ and *v*_*k*_ from *G*.

*Closeness* centrality is defined as the inverse of the sum of geodesic distances to all other nodes in *G*^1,56^, and can be considered as a measure of how long it will take to spread information from a given node to other reachable nodes in the network:

6 $$Cls({v}_{i})={(\sum _{{v}_{j}\in G\backslash {v}_{i}}d({v}_{i},{v}_{j}))}^{-1}$$

where *d*(*v*_*i*_, *v*_*j*_) is the distance (number of hops) between the two nodes *v*_*i*_ and *v*_*j*_.

The most common centrality based on the random walk process is the *Eigenvector* centrality (*EC*), which assumes that the influence of a node is not only determined by the number of its neighbors, but also by the influence of each neighbor^23^. The centrality of any node is proportional to the sum of neighboring centralities^1^. Considering a constant *λ*, the *EC* is formally defined as:

7 $$EC({v}_{i})=\frac{1}{\lambda }\sum _{{v}_{j}\in {V}_{i}}EC({v}_{j})$$

### Assessing network fidelity

In order to assess the structural realism of the generated social networks, we used the *statistical fidelity φ*, which is proven to offer reliable insights on complex network topologies^25^. The fidelity metric *φ* numerically captures the similarity between any graph topology G^*^ with respect to another reference graph *G* (*i.e*., a complex network *G* = (*V*, *E*)). More precisely, by measuring and comparing their common individual graph metrics, a maximum fidelity of 1 represents complete similarity, while a minimum fidelity of 0 represents complete dissimilarity between the two compared topologies. Of note, the fidelity is *not* dependent on the choice of metrics of interest, however it is customizable to allow a weighted comparison. Depending on the context of the problem, any numerical value (*i.e*. metric) that is representative for the model can be used. The definition and proof of statistical fidelity *φ* are detailed in^25^.

**Definition 1**. Given a reference topology *G*, and any other network G^*^ being compared to *G*, the arithmetic fidelity ${\phi }_{A}^{\ast }$, which expresses the similarity between G^*^ and *G*, is defined as:

8 $${{\phi }}_{A}^{\ast }=\{\begin{array}{ll}\frac{1}{n}\,\sum _{i=1}^{n}\,\frac{{m}_{i}}{2{m}_{i}-{m}_{i}^{\ast }} & if\,{m}_{i}^{\ast } < {m}_{i},\,{m}_{i}=0\\ \frac{1}{n}\,\sum _{i=1}^{n}\,\frac{{m}_{i}}{{m}_{i}^{\ast }} & if\,{m}_{i}^{\ast }\ge {m}_{i},\,{m}_{i}=0\\ \frac{1}{n}\,\sum _{i=1}^{n}\,\frac{1}{{m}_{i}^{\ast }+1} & if\,{m}_{i}=0\end{array}$$

In equation 8, *i* is the index of the metric which describes the two networks being compared, and *n* is the total number of metrics used in the comparison. In this paper we compute the fidelity between multiple synthetic topologies and the empirical social network references. These reference datasets are chosen because they have typical real-life social network features. The fidelity comparison is made relative to the set of relevant network metrics (indexed by *i*).

In this paper, fidelity is measured by taking into consideration the following topological characteristics: average degree *AD*, average path length *APL*, average clustering coefficient *ACC*, modularity *Mod*, diameter *Dmt*, and density *Dns*.

## Electronic supplementary material

Supplementary Information
