# Supplementary material for: Weighted Betweenness Preferential Attachment: A New Mechanism Explaining Social Network Formation and Evolution
Source: Sci Rep. 2018 Jul 18;8:10871. doi: 10.1038/s41598-018-29224-w (PMC6052171; doi:10.1038/s41598-018-29224-w)
Supplement: Supplementary file 1 — Supplementary Information [file 41598_2018_29224_MOESM1_ESM.pdf]

Supplementary Information

Weighted Betweenness Preferential Attachment: A New Mechanism Explaining Social Network Formation and Evolution

Alexandru Topîrceanu, Mihai Udrescu, and Radu Mărculescu

SI.1. Social network datasets statistics

Social networks can be characterized by their specific node degree distribution function $P < k >$ which normally follows a power-law like distribution[1]. As such, we present in Table 1 the γ distribution slopes of node degree, node betweenness, and link weight for our empirical datasets. These results are obtained using the *poweRlaw* package available in *R*. The averaged slopes are 2.097 ± 0.774 for degree, 1.609 ± 0.431 for betweenness, and 2.987 ± 1.141 for edge weight; thus degree distribution is 30.3% steeper than betweenness distribution.

Table 1. Power-law interpolation slopes γ for the distribution of node degree (deg), node betweenness (btw), and link weight for real-world datasets, obtained using the *poweRlaw* package in *R*.

Dataset	Node-deg γ	Node-btw γ	Link-weight γ
Facebook	1.375	1.137	-
Google Plus	1.301	1.149	-
Co-authorships	2.519	2.055	1.566
Online SN	1.486	1.177	2.574
Bitcoin OTC	2.102	1.856	5.052
Wiki-Votes	3.870	2.335	-
Geom	2.145	1.392	3.760
CondMat	3.452	2.155	-
MathOverflow	1.810	1.652	3.152
POK	1.815	1.135	2.567
Email Enron	1.958	1.871	-
Brightkite OSN	2.505	2.005	-

Statistical analysis of betweenness-degree relationship in empirical social networks shows a non-linear dependency between the two node centralities, namely betweenness distribution has a polynomial or exponential rise in relation to degree distribution taken over the same nodes. This aspect is detailed in Figure 1. Each panel contains the best approximating interpolation function for the correlation betweenness-degree.

In order to analyze whether there is a natural attraction between nodes with high fitness (specifically degree and betweenness) and links weights, we use a Pareto approach. Theories like the "80/20" principle[1], the "distribution of wealth" (e.g. 10% of the people own 90% of the wealth)[7], are all examples in which there is no direct, linear correlation between a node's property (cause) and it's contextual value (effect), but we can observe associations when we divide a population in percentiles. In an analogous manner, we systematically filter our weighted datasets by leaving the top 1, 2, 5, 10, 25, 50, 75 and 100% percentiles in terms of link weights. For each percentile, we measure the accumulated fitness and weight. These associations are quantified in Table 2 and depicted in Figure 2.

The association methodology implies summing up the weights on all incident links for every node in the network, and then correlating each sum with the measured fitness of the node.

Table 2. Accumulated weight, degree and betweenness (btw) in the top percentiles of nodes filtered by highest link weight. The values are normalized based on the total amount of fitness in each graph, thus they can be interpreted as percentages.

	Accumulated *degree* in percentile								Accumulated *betweenness* in percentile							
**Dataset**	1	2	5	10	25	50	75	100	1	2	5	10	25	50	75	100
Co-authorships	0.049	0.082	0.149	0.238	0.468	0.666	0.825	1	0.477	0.623	0.802	0.943	0.999	1	1	1
Online SN	0.1	0.172	0.317	0.488	0.761	0.94	0.988	1	0.253	0.366	0.563	0.716	0.907	0.983	1	1
Geom	0.134	0.198	0.310	0.429	0.633	0.823	0.935	1	0.351	0.468	0.639	0.766	0.931	0.991	1	1
POK	0.174	0.245	0.383	0.525	0.749	0.902	0.966	1	0	0	0.074	0.12	0.394	0.777	1	1

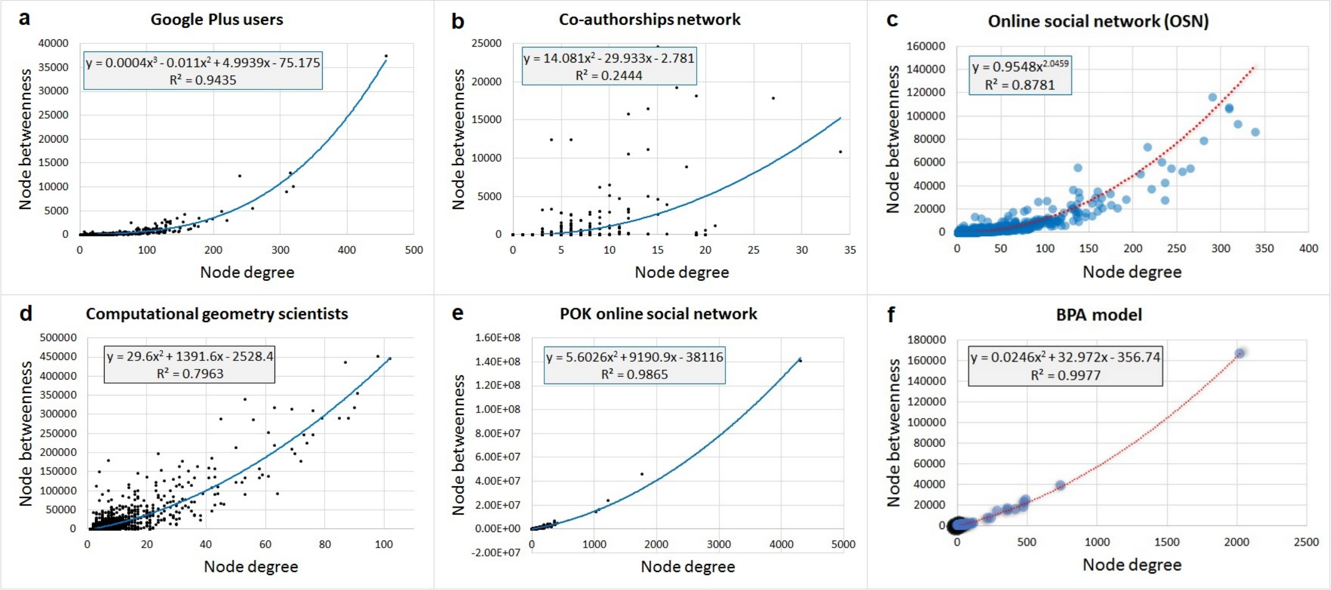

Figure 1. Non-linear dependency of node betweenness and degree in empirical social networks characterized by polynomial interpolation functions. **a.** Google Plus users network[2] (638 nodes). **b.** Weighted co-authorships in network science[3] (1589 nodes). **c.** Weighted online social network[4] (1899 nodes). **d.** Geom network[5] (7343 nodes). **e.** Weighted POK online user network[6] (29K nodes). **f.** Proposed BPA network model (10K nodes).

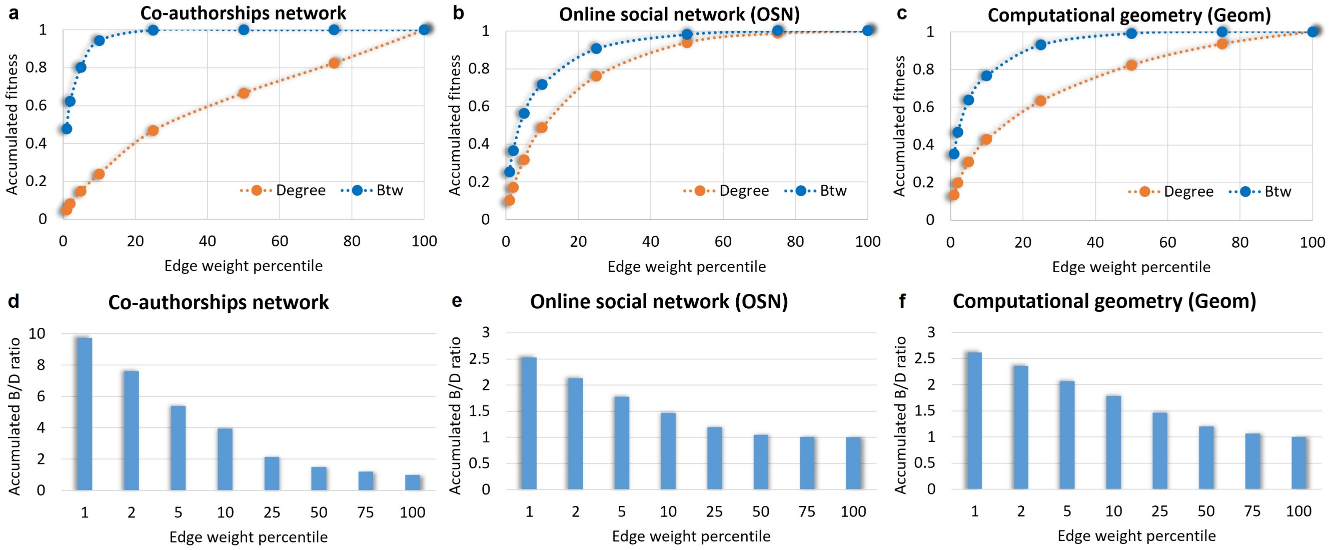

Figure 2. Accumulated degree and betweenness (Btw) in each percentile of nodes filtered by highest link weights. The lower panel depicts the computed ratio Btw/Degree from the upper panel values, highlighting the increased association of betweenness with link weights.

Based on the statistics of betweenness, we argue that the betweenness/degree (B/D) ratio in a social network should have a uniform distribution. As such, Figure 3 presents how the B/D ratios are centered around the value of B/D = 1, in the interval (0.1, 1000). It can be observed that all empirical data, weighted or unweighted, has a specific distribution pattern which presents an even distribution in the selected interval. However, all synthetic datasets (random, small-world, scale-free) used for null-model validation have a considerably narrow interval for the B/D ratio.

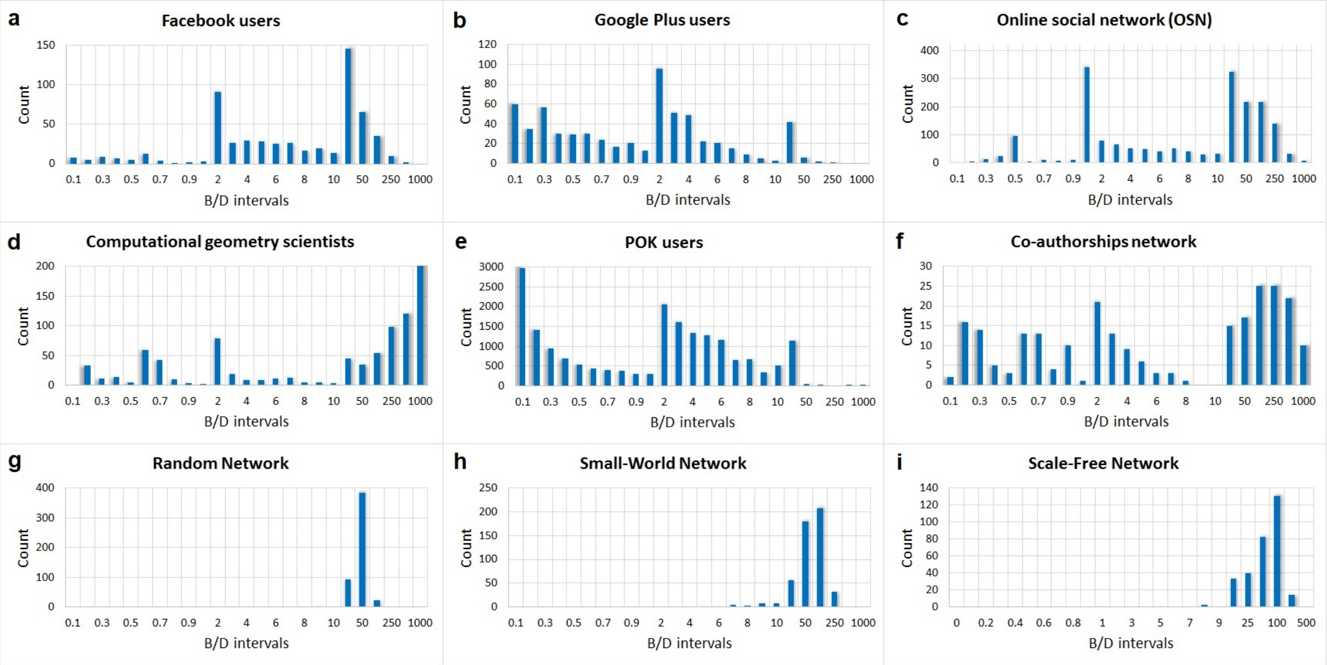

Figure 3. Distributions of betweenness/degree (B/D) ratios in empirical and synthetic social networks characterized by Gini coefficients g. **a.** Facebook users network[8] ($g_{FB} = 0.5955$). **b.** Google Plus users network[2] ($g_{GP} = 0.4820$). **c.** Online social network[4] ($g_{OSN} = 0.5921$). **d.** Scientifc collaboration network[5] ($g_{Geom} = 0.610$). **e.** Weighted POK online user network[6] ($g_{POK} = 4879$). **f.** Co-authorships network[3] ($g_{CoAu} = 0.4392$). **g.** Random network[9] ($g_{rand} = 0.9374 \pm 0.0013$) **h.** Small-world network[10] ($g_{SW} = 0.8771 \pm 0.0451$). **i.** DPA network[1] ($g_{DPA} = 0.7828 \pm 0.0182$).

SI.2. Best fitness for preferential attachment

The realism assessment based on the comparison between the synthetic preferential attachment (PA) networks and the real-world datasets is done through individual graph metric comparison, as well as using the composite statistical fidelity metric[11]. First, we analyze the distribution of the following six graph metrics on the real-world datasets: average degree (AD), average path length (APL), average clustering coefficient (ACC), modularity (Mod), graph diameter (Dmt), and graph density (Dns). The evolution of these metrics from network sizes $N = 590$ to $N = 364K$ nodes is presented in Figure 4.

The diversity of the datasets is high, so the purpose of the trend lines present in Figure 4 is not to suggest that graph metrics abide a strict confidence interval, but rather to show how each graph metric evolves with increasing network size (*i.e.*, from $N = 590$ to $N = 364K$). As such, we notice the following trends:

- AD increases from 10 ($N \approx 1,000$) to 25 ($N \approx 100,000$), within $AD \approx [3-33]$ and standard deviation $\sigma = 8.904$.
- APL increases slowly from 4 ($N \approx 1,000$) to 4.5 ($N \approx 100,000$), within $AP \approx [2.5-5.8]$ and $\sigma = 0.901$.
- ACC decreases from 0.5 ($N \approx 1,000$) to 0.1 ($N \approx 100,000$), within $ACC \approx [0.02-0.88]$ and $\sigma = 0.251$.
- Mod remains stable within around 0.5, but has a high variation depending on the nature of the dataset within $Mod \approx [0.25-0.78]$ and $\sigma = 0.156$.
- Dmt increases from 7 ($N \approx 1,000$) to 14 ($N \approx 100,000$), within $Dmt \approx [7-17]$ and $\sigma = 2.948$.
- Dns drops to 0 for large datasets, from roughly 0.005 ($N \approx 1,000$) to 0 ($N \approx 100,000$), within $Dns \approx [0-0.008]$ and $\sigma = 0.002$.

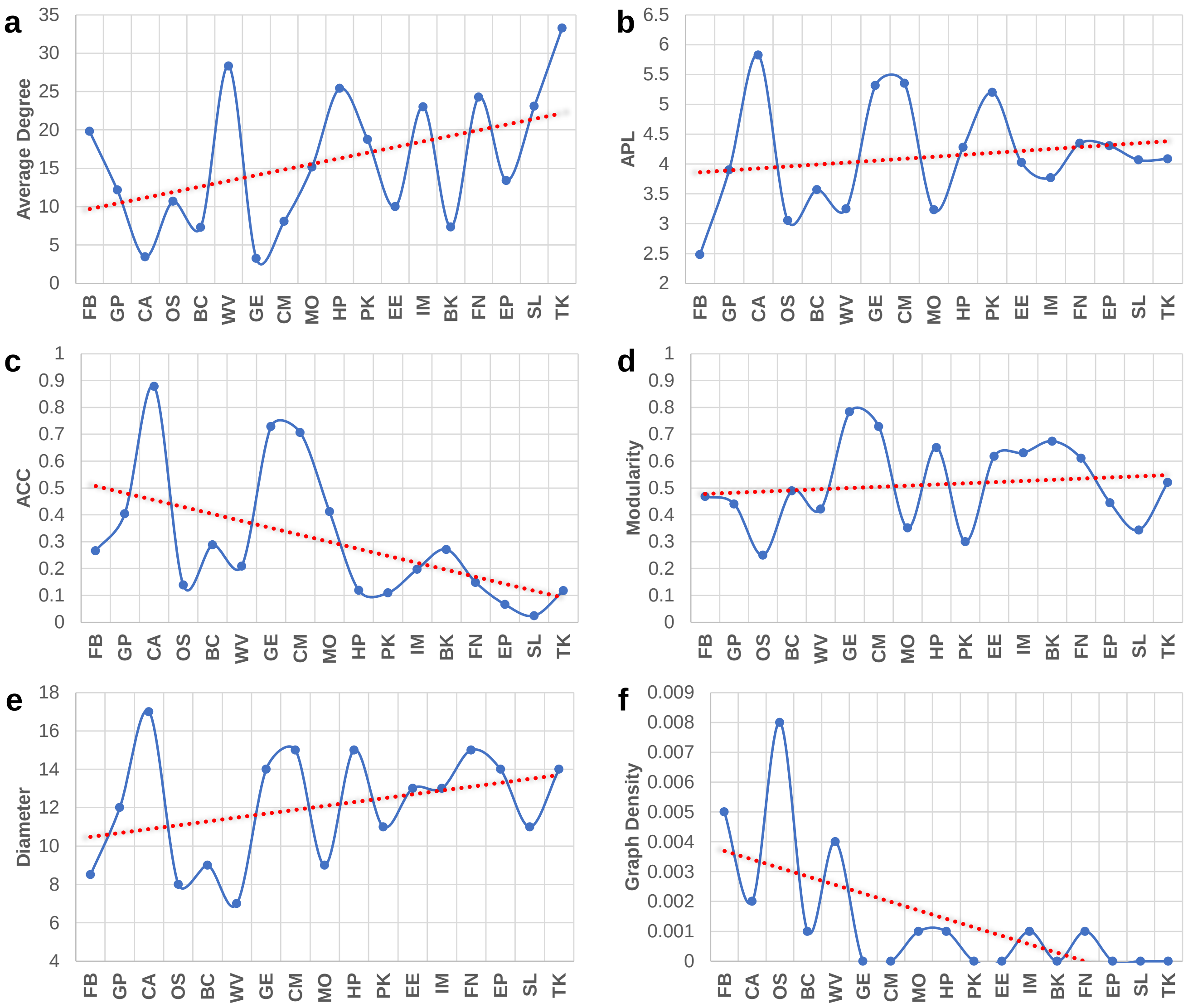

Figure 4. Distribution of the six fundamental graph metrics (a-f) over the 18 validation datasets (refer to Table 5 in the main manuscript for acronyms). Datasets on the OX axis are ordered by increasing network size, from N=590 to N=364K nodes. The trend line is suggested with a red dotted line.

Having established a general overview of how graph metrics evolve in real data based on network size, we further generate WPBA (based on betweenness Btw as node fitness) networks and compare them to DPA (based on degree Deg), ECPA (based on eigenvector centrality EC), ClsPA (based on closeness Cls) and CCPA (based on clustering coefficient CC). To have a comparison base with the real-world results in Figure 4, we make measurements and then average them for 10 networks corresponding to each centrality, for network sizes of $N = 1K, 2K, 5K, 10K, 50K, 100K$ nodes. In Figure 5 we represent the evolution of the same six graph metrics in preferential attachment (PA) networks. To keep the visualization intuitive, we highlight with error bars the minimum and maximum measurements only for DPA and WBPA networks. We also suggest, with a dotted orange line, the corresponding metric evolution for the random networks that we use as a *Null* model. The random networks are generated with the same number of nodes and average degree as the corresponding PA networks.

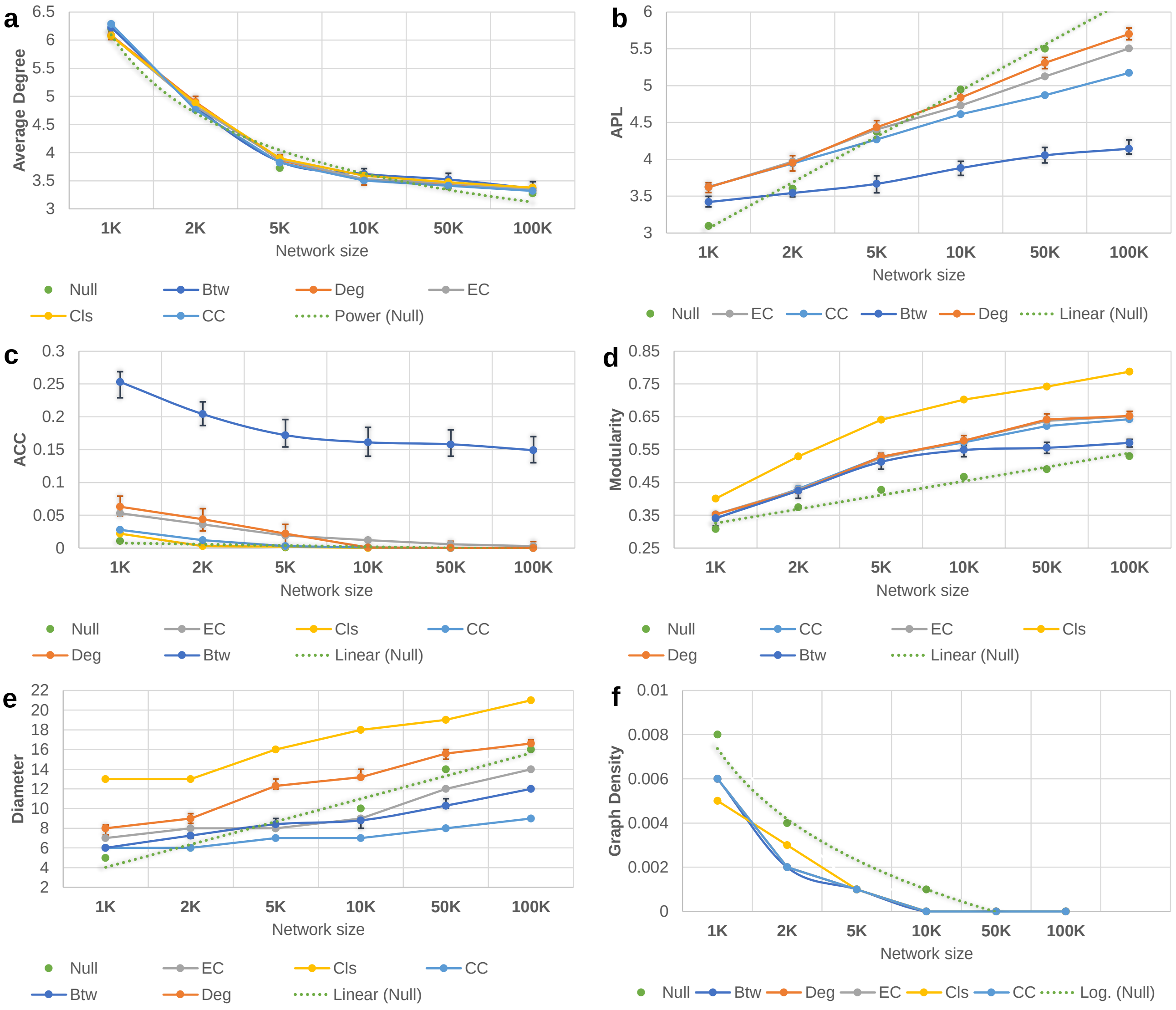

Figure 5. Distribution of the six fundamental graph metrics (a-f) for synthetic datasets generated according to the preferential attachment principle using as node fitness: betweenness (*Btw*), degree (*Deg*), eigenvector centrality (*EC*), closeness (*Cls*), and clustering coefficient (*CC*). The orange dotted line represents the trend line obtained for similar sized random networks (used as a *Null* model). The plots for *Deg* (red) and *Btw* (blue) contain error bars, highlighting the min-max values for each set of measurements.

By analyzing solely the synthetic results, we conclude that:

- *AD* drops from 6.2 to 3.3 for all networks as network size N increases.
- *APL* increases slowly, and *Btw* generates a much lower *APL* than all the other centralities. *Cls* is omitted from the figure because it is placed much higher ($AD \approx [5-8]$), above the focused area. The *APL* of *Btw* networks increases from 3.4 ± 0.08 ($\sigma = 0.046$) ($N \approx 1,000$) to 4.1 ± 0.12 ($\sigma = 0.056$) ($N \approx 100,000$), with an average standard deviation of $\sigma = 0.0561$. The *APL* of all other PA networks increases from around 3.6 ($N \approx 1,000$) to 5.2 ($N \approx 100,000$).
- *ACC* decreases for all datasets, however this is a notable difference between *Btw* and all other networks. Indeed, *Btw* networks (WBPA) are the only ones capable of producing significant clustering in the network, starting from 0.25 ± 0.016 ($N \approx 1,000$) down to 0.15 ± 0.011 ($N \approx 100,000$). The other networks start at around 0.05 ± 0.016 ($N \approx 1,000$) and decrease to 0 ($N \approx 100,000$).

- *Mod* increases and converges to distinct values for each centrality. *Cls* produces very modular structures for large networks ($Mod \approx [0.4 - 0.8]$). *Btw* creates average modular structures, with $Mod \approx [0.34 \pm 0.023 - 0.57 \pm 0.013]$ and converges towards values < 0.6. On the other hand, *Deg*, *CC* and *EC* produce stronger community structure for large networks, increasing from 0.35 ± 0.016 ($N \approx 1,000$) to 0.65 ± 0.014 ($N \approx 100,000$).

- *Dmt* increases for all networks, yet every single centrality has its own characteristic increase. In general, the *Dmt* is similar to that of the null model which increases from 5 ($N \approx 1,000$) to 16 ($N \approx 100,000$). *CC* and *Btw* produce the shortest diameter networks, while *Cls* and *Deg* produce the longest diameters.

- *Dns* decreases for all networks in a similar manner, from 0.005 ($N \approx 1,000$) to 0 ($N \approx 100,000$).

SI.3. UPT-Social: an emerging online social network use case

The objective of this section is to briefly present empirical evidence that node betweenness is a better centrality than node degree in terms of attracting new social ties. In this context, we use a dataset with timestamped dynamical data[12]. *UPT-social* captures the birth (launch), and initial growth, over 6 weeks of a newly launched online social platform. The dataset contains detailed data about each node over multiple snapshots in time, and reaches a size of 351 users in 44 days after launch.

The UPT-social dataset provides several snapshots at relative moment in time after launch (day 0), namely: days 3, 7, 15, 24, and 44. For simplicity, we refer to these moments in time as T_{1-5}. For each T_i we define a weighted correlation function, for both node centralities, called link attractiveness α, as:

$$\alpha_D(G) = \sum_{v_i \in G} (k_i^* \cdot r_i^*), \qquad \alpha_B(G) = \sum_{v_i \in G} (b_i^* \cdot r_i^*) \tag{1}$$

where k_i is the degree of a node v_i, respectively b_i its betweenness; r_i is the number of received (attracted) new links in T_i from T_{i-1}. The * superscript represents the fact that all three metrics (k_i, b_i and r_i) are normalized. The obtained α-sums are given in Table 3, and represent weighted correlations between a node's centrality and it's ability to attract new links, summed up over the whole network. We obtain consistent results that betweenness has a higher attractiveness than degree, within a range of roughly +6-49%.

Table 3. Evoltion of degree (k) and betweenness (b) attractiveness α in the UPT-social dataset over 4 moments T_i in time.

	α_k	α_b	$\alpha_b/\alpha_k(\%)$
T_2	0.01265	0.01478	+16.8%
T_3	0.00907	0.00958	+5.6%
T_4	0.00843	0.01255	+48.8%
T_5	0.01334	0.01803	+35.1%

A second analysis we present studies the evolution and correlation in time of the same three metrics: degree, betweenness and received links. We obtain consistent results for most relevant nodes in the network, and present them in Figure 6 for the top 3 nodes, in terms of received links, for snapshots T_{3-5}. The left-most vertical panel in Figure 6 corresponds to the *oldest* receivers, which were in the top 3 during $T2$; these nodes present the full evolution of betweenness: rise, peak, fall and stabilization; respectively rise and saturation of degree, as explained through the social evolution cycle in the *Discussion* section (Figure 7). The middle and right panels of Figure 6 represent similar evolutions of the the two centralities, but capture the nodes in their middle ages of evolution.

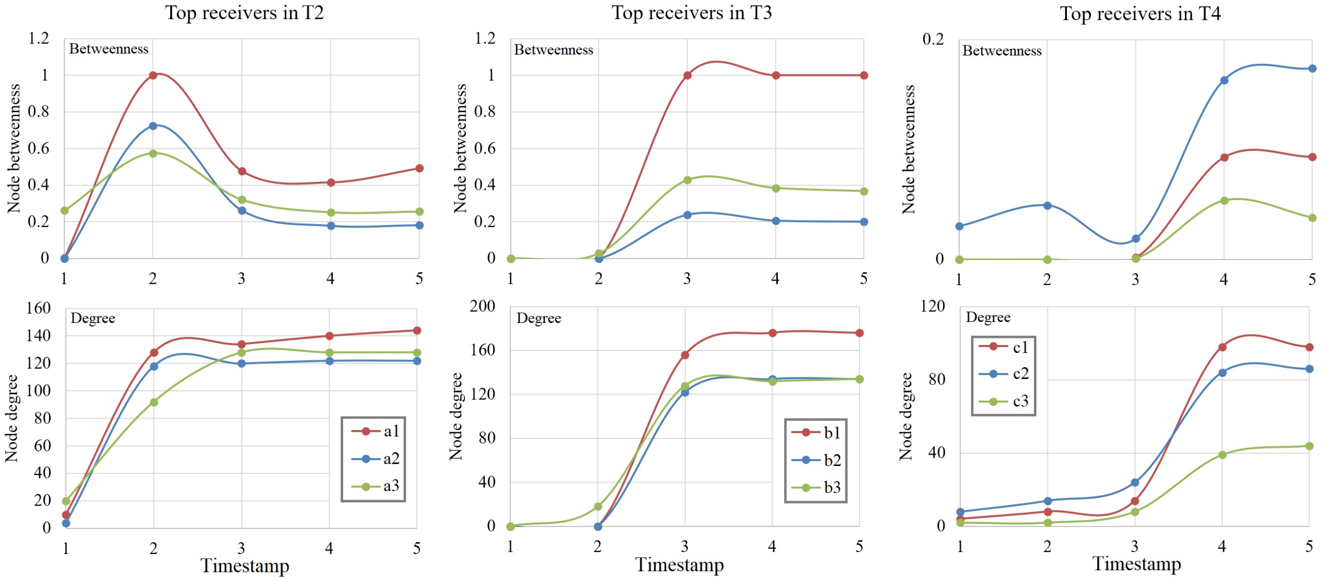

Figure 6. Comparative evolution in time for betweenness and degree on the nodes which were the top 3 link receivers, as measured in T_2, T_3 and T_4. The names of the nodes (e.g. *a1, b2, c3*) are anonymized, and are used solely for identification.

Both numerical and visual results are presented to support the higher potential of betweenness centrality as a driver in social network emergence, and further explain the social evolution cycle – and implicit degree-betweenness dependency – we propose in the paper.

SI.4. State of the art network model comparison

In order to extend our realism assessment of WBPA, we introduce several social network models, approved in the field, as a comparison baseline. As such, we make use of the Cellular[13], Holme-Kim[14], Toivonen[15], and WSDD[16] networks. Their averaged metric measurements are presented in Table 4, alongside the standard deviations ($\pm\sigma$) after generating 10 networks of size $N = 10,000$ nodes for each model.

Cellular networks are composed out of semi-independent cells of small sizes, each with one node acting as a cell leader. Only leaders may connect to other cells, via their respective leaders, resulting in a highly decentralized topology. Inspired by covert networks, this is a non-traditional organizational configuration, without a hierarchical structure.

The Holme-Kim model extend the BA scale-free network model[1] to include a triad formation step. A high degree of realism results from using this model because it possesses the same characteristics as the standard scale-free networks, such as power-law degree distribution and small average path length, but adds a high clustering at the same time.

The Toivonen model starts from a different perspective, namely that real-world social networks are divided into communities with dense internal connections, resulting in consistently high values of the clustering coefficient. Also, the authors consider the observed degree assortativity, and the broad degree distribution. The Toivonen model is capable of reproducing realistic synthetic networks based on a mixture of random attachment and implicit preferential attachment.

Finally, the Watts-Strogatz model with degree distribution (WSDD) is a small-world network with enhanced preferential attachment. The creation algorithm relies on generating a set of independent communities inside which preferential attachment is applied; then, each community is connected to other communities, as if they were unconnected nodes, with the Watts-Strogatz algorithm.

Table 4. Mean values of average degree (*AD*), average path length (*APL*), average clustering coefficient (*ACC*), modularity (*Mod*), diameter (*Dmt*), and density (*Dns*) averaged ($\pm$ standard deviation σ) for synthetic state of the art network models of size $N = 10,000$ nodes.

Dataset	*AD*	*APL*	*ACC*	*Mod*	*Dmt*	*Dns*
Small-world	3.99 ± 0.138	5.61 ± 0.325	0.321 ± 0.012	0.730 ± 0.021	11 ± 0.50	0.005 ± 0.003
Cellular	11.39 ± 0.269	3.79 ± 0.227	0.599 ± 0.023	0.910 ± 0.016	7 ± 0.50	0.020 ± 0.003
Holme-Kim	3.37 ± 0.161	3.40 ± 0.210	0.503 ± 0.008	0.489 ± 0.009	7 ± 1.00	0.011 ± 0.002
Toivonen	2.09 ± 0.101	4.89 ± 0.149	0.648 ± 0.015	0.796 ± 0.018	10 ± 0.50	0.060 ± 0.002
WSDD	21.58 ± 0.404	4.59 ± 0.103	0.738 ± 0.041	0.900 ± 0.020	9 ± 0.00	0.041 ± 0.002

The described state of the art network models are compared alongside WBPA, DPA and the random network model, with multiple real-world datasets in terms of their ability to reproduce similar graph metrics. The comparison is expressed using the fidelity metric ϕ, and is available in the main manuscript in Table 3. Table 5 provides the averaged measures for the WBPA, DPA and random network models, for graphs of size $N = 10,000$ nodes.

Table 5. Mean values of average degree (AD), average path length (APL), average clustering coefficient (ACC), modularity (Mod), diameter (Dmt), and density (Dns) averaged ($\pm$ standard deviation σ) for the WBPA, DPA, and random network models of size $N = 10,000$ nodes.

Dataset	*AD*	*APL*	*ACC*	*Mod*	*Dmt*	*Dns*
WBPA-10K	3.621 ± 0.022	3.881 ± 0.049	0.161 ± 0.011	0.549 ± 0.012	8.8 ± 0.238	0 ± 0.000
DAP-10K	3.527 ± 0.046	4.835 ± 0.064	0.001 ± 0.000	0.577 ± 0.008	13.2 ± 0.304	0 ± 0.000
Rand-10K	3.509 ± 0.038	4.946 ± 0.045	0.001 ± 0.000	0.467 ± 0.003	10 ± 0.000	0.001 ± 0.000

SI.5. Datasets availability

In Table 6 we detail the information about the nature of nodes and links, as well as direct URLs for acquiring each dataset used in this paper for real-world accuracy test.

Table 6. Node and link details, as well as repository URL for each empirical dataset (the unweighted datasets are listed in the upper part, while the weighted datasets are listed in the lower part of the table). Larger numerical values are expressed as thousands (K) or millions (M).

Dataset	# Nodes	*Node nature*	# Links	*Link nature*
Facebook	590	Facebook users	5847	On-line friendship (unweighted)<br>*https://staff.cs.upt.ro/ alext/acsanet*
Google Plus	638	Google Plus users	3875	On-line friendship (unweighted)<br>*https://snap.stanford.edu/data/egonets-Gplus.html*
Wikipedia votes	7115	Wikipedia users	101K	Vote for website administrators (unweighted)<br>*https://snap.stanford.edu/data/wiki-Vote.html*
CondMat collaboration	23K	CondMat scientists	93K	Collaboration on Arxiv in *Condensed Matter* (unweighted)<br>*https://snap.stanford.edu/data/ca-CondMat.html*
HEP citations	28K	HEP scientists	353K	Citations between *High Energy Particle Physics* scientists (unweighted)<br>*http://vlado.fmf.uni-lj.si/pub/networks/data/hep-th/hep-th.htm*
Email Enron	37K	Enron email users	184K	Email communication from Enron (unweighted)<br>*https://snap.stanford.edu/data/email-Enron.html*
IMDB co-appearances	48K	Adult movie actors	1.1M	Co-appearances in the same *adult* movie on IMDB (unweighted)<br>*http://www-levich.engr.ccny.cuny.edu/webpage/hmakse/software-and-data/*
Brightkite OSN	58K	Brightkite users	214K	On-line friendship (unweighted)<br>*https://snap.stanford.edu/data/loc-brightkite.html*
Facebook New-Orleans	64K	Facebook users from New Orleans	1.5M	On-line friendship (unweighted)<br>*http://socialnetworks.mpi-sws.org/data-wosn2009.html*
Epinions social network	76K	Members on epinions.com	508K	On-line friendship (unweighted)<br>*https://snap.stanford.edu/data/soc-Epinions1.html*
Slashdot social network	82K	Slashdot users	948K	On-line friendship (unweighted)<br>*https://snap.stanford.edu/data/#socnets*
Co-authorships	1589	Scientific authors	2742	Network science co-authorships (weighted)<br>*https://github.com/gephi/gephi/wiki/Datasets*
Online social network	1899	Social network platform users	20296	On-line friendship intensity (weighted)<br>*https://github.com/gephi/gephi/wiki/Datasets*
Bitcoin OTC	5881	Bitcoin users	21492	Trust (reputation) between two users (weighted)<br>*https://snap.stanford.edu/data/soc-sign-bitcoinotc.html*
Geom collaboration	7343	Computational geometry scientists	11898	Scientific collaboration (weighted)<br>*http://vlado.fmf.uni-lj.si/pub/networks/data/collab/geom.htm*
MathOverflow	25K	MathOverflow users	188K	Interactions on Math Overflow website (weighted)<br>*https://snap.stanford.edu/data/sx-mathoverflow.html*
POK social network	29K	POK users	115K	Private messages (weighted)<br>*http://www-levich.engr.ccny.cuny.edu/webpage/hmakse/software-and-data/*
Timik platform	364K	Timik.pl on-line players	6069K	Number of chats between players (weighted)<br>*http://dx.doi.org/10.7910/DVN/V6AJRV*

References

1. Barabási, A.-L. & Albert, R. Emergence of scaling in random networks. *science* **286**, 509–512 (1999).

2. McAuley, J. J. & Leskovec, J. Learning to discover social circles in ego networks. In *NIPS*, vol. 2012, 548–56 (2012).
3. Newman, M. E. Finding community structure in networks using the eigenvectors of matrices. *Phys. review E* **74**, 036104 (2006).
4. Opsahl, T. & Panzarasa, P. Clustering in weighted networks. *Soc. networks* **31**, 155–163 (2009).
5. Batagelj, V. & Mrvar, A. Pajek datasets (2006).
6. Takac, L. & Zabovsky, M. Data analysis in public social networks. In *International Scientific Conference and International Workshop Present Day Trends of Innovations*, 1–6 (2012).
7. Levy, M. & Solomon, S. New evidence for the power-law distribution of wealth. *Phys. A: Stat. Mech. its Appl.* **242**, 90–94 (1997).
8. Topirceanu, A., Udrescu, M. & Vladutiu, M. Genetically optimized realistic social network topology inspired by facebook. In *Online Social Media Analysis and Visualization*, 163–179 (Springer, 2014).
9. Wang, X. F. & Chen, G. Complex networks: small-world, scale-free and beyond. *Circuits Syst. Mag. IEEE* **3**, 6–20 (2003).
10. Strogatz, S. H. Exploring complex networks. *Nat.* **410**, 268–276 (2001).
11. Topirceanu, A. & Udrescu, M. Statistical fidelity: a tool to quantify the similarity between multi-variable entities with application in complex networks. *Int. J. Comput. Math.* 1–19 (2016).
12. Topirceanu, A., Garcia, J. & Udrescu, M. Upt. social: The growth of a new online social network. In *Network Intelligence Conference (ENIC), 2016 Third European*, 9–16 (IEEE, 2016).
13. Tsvetovat, M. & Carley, K. M. Generation of realistic social network datasets for testing of analysis and simulation tools. Tech. Rep., DTIC Document (2005).
14. Holme, P. & Kim, B. J. Growing scale-free networks with tunable clustering. *Phys. Rev. E* **65**, 026107 (2002).
15. Toivonen, R., Onnela, J.-P., Saramäki, J., Hyvönen, J. & Kaski, K. A model for social networks. *Phys. A: Stat. Mech. its Appl.* **371**, 851–860 (2006).
16. Chen, Y., Zhang, L. & Huang, J. The watts–strogatz network model developed by including degree distribution: theory and computer simulation. *J. Phys. A: Math. Theor.* **40**, 8237 (2007).
